# QRS micro-fragmentation as a mortality predictor^[Author-notes ehac085-FM1]^

**DOI:** 10.1093/eurheartj/ehac085

**Published:** 2022-02-21

**Authors:** Katerina Hnatkova, Irena Andršová, Tomáš Novotný, Annie Britton, Martin Shipley, Bert Vandenberk, David J Sprenkeler, Juhani Junttila, Tobias Reichlin, Simon Schlögl, Marc A Vos, Tim Friede, Axel Bauer, Heikki V Huikuri, Rik Willems, Georg Schmidt, Michael R Franz, Christian Sticherling, Markus Zabel, Marek Malik

**Affiliations:** National Heart and Lung Institute, Imperial College, ICTEM, Hammersmith Campus, 72 Du Cane Road, Shepherd’s Bush, London W12 0NN, UK; Department of Internal Medicine and Cardiology, University Hospital Brno, Brno, Czech Republic; Department of Internal Medicine and Cardiology, Masaryk University, Brno, Czech Republic; Department of Internal Medicine and Cardiology, University Hospital Brno, Brno, Czech Republic; Department of Internal Medicine and Cardiology, Masaryk University, Brno, Czech Republic; Research Department of Epidemiology and Public Health, University College London, UK; Research Department of Epidemiology and Public Health, University College London, UK; Department of Cardiovascular Sciences, University of Leuven, Leuven, Belgium; Department of Medical Physiology, University Medical Center Utrecht, Utrecht, The Netherlands; Medical Research Center Oulu, University Central Hospital of Oulu and University of Oulu, Oulu, Finland; Department of Cardiology, Inselspital, Bern University Hospital, Bern, Switzerland; Department of Cardiology and Pneumology, University Medical Center, Göttingen, Germany; German Center of Cardiovascular Research (DZHK), Partner Site Göttingen, Göttingen, Germany; Department of Medical Physiology, University Medical Center Utrecht, Utrecht, The Netherlands; German Center of Cardiovascular Research (DZHK), Partner Site Göttingen, Göttingen, Germany; Department of Medical Statistics, University Medical Center Göttingen, Göttingen, Germany; University Hospital for Internal Medicine III, Medical University Innsbruck, Innsbruck, Austria; Medical Research Center Oulu, University Central Hospital of Oulu and University of Oulu, Oulu, Finland; Department of Cardiovascular Sciences, University of Leuven, Leuven, Belgium; Klinikum rechts der Isar, Technical University of Munich, Munich, Germany; German Center for Cardiovascular Research Partner Site Munich Heart Alliance, Munich, Germany; Veteran Affairs and Georgetown University Medical Centers, Washington, DC, USA; Department of Cardiology, University Hospital of Basel, Basel, Switzerland; Department of Cardiology and Pneumology, University Medical Center, Göttingen, Germany; German Center of Cardiovascular Research (DZHK), Partner Site Göttingen, Göttingen, Germany; National Heart and Lung Institute, Imperial College, ICTEM, Hammersmith Campus, 72 Du Cane Road, Shepherd’s Bush, London W12 0NN, UK; Department of Internal Medicine and Cardiology, Masaryk University, Brno, Czech Republic

**Keywords:** Electrocardiogram, QRS complex, Fragmentation, Mortality prediction

## Abstract

**Aims:**

Fragmented QRS complex with visible notching on standard 12-lead electrocardiogram (ECG) is understood to represent depolarization abnormalities and to signify risk of cardiac events. Depolarization abnormalities with similar prognostic implications likely exist beyond visual recognition but no technology is presently suitable for quantification of such invisible ECG abnormalities. We present such a technology.

**Methods and results:**

A signal processing method projects all ECG leads of the QRS complex into optimized three perpendicular dimensions, reconstructs the ECG back from this three-dimensional projection, and quantifies the difference (QRS ‘micro’-fragmentation, QRS-*μf*) between the original and reconstructed signals. QRS ‘micro’-fragmentation was assessed in three different populations: cardiac patients with automatic implantable cardioverter-defibrillators, cardiac patients with severe abnormalities, and general public. The predictive value of QRS-*μf* for mortality was investigated both univariably and in multivariable comparisons with other risk factors including visible QRS ‘macro’-fragmentation, QRS-*Mf*. The analysis was made in a total of 7779 subjects of whom 504 have not survived the first 5 years of follow-up. In all three populations, QRS-*μf* was strongly predictive of survival (*P* < 0.001 univariably, and *P* < 0.001 to *P* = 0.024 in multivariable regression analyses). A similar strong association with outcome was found when dichotomizing QRS-*μf* prospectively at 3.5%. When QRS-*μf* was used in multivariable analyses, QRS-*Mf* and QRS duration lost their predictive value.

**Conclusion:**

In three populations with different clinical characteristics, QRS-*μf* was a powerful mortality risk factor independent of several previously established risk indices. Electrophysiologic abnormalities that contribute to increased QRS-*μf* values are likely responsible for the predictive power of visible QRS-*Mf*.

## Structured Graphical Abstract

**Structured Graphical Abstract:**
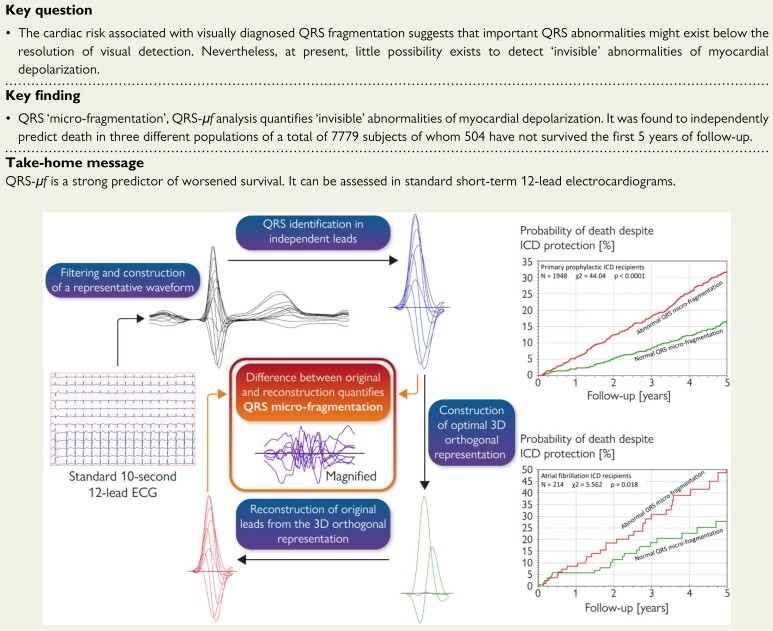
Principles of QRS micro-fragmentation.

**See the editorial comment for this article ‘The fractionated QRS complex for cardiovascular risk assessment’, by Richard N. W. Hauer, https://doi.org/10.1093/eurheartj/ehac198.**

## Introduction

Abnormalities of the electrocardiographic QRS complex reflect intramyocardial conduction pathologies. Such abnormalities include not only the typical bundle branch block patterns but also forms due to less specific intraventricular conduction abnormalities. Prolonged QRS complex duration has long been a recognized risk factor for adverse cardiac events.^1,2^ More recently, the so-called QRS complex fragmentation, defined by visually detected splits of QRS waves,^3,4^ has also been found to predict poor outcome in both cardiac patients and other well-defined groups^5–7^ including the general population.^4^ This increased risk due to visible QRS fragmentation appears independent of the overall QRS complex duration.^8^

Visual diagnosis of QRS fragmentation leads to a yes/no classification although quantitative sums of QRS splits detected in different leads and probabilistic approaches have also been proposed.^9^ This categorical distinction suggests that similarly important QRS abnormalities might exist below the resolution of visual detection. Such a concept is not new. Already some decades ago, spectral analyses of signal-averaged QRS complex were proposed, albeit with variable success, to identify abnormalities hidden within the overall QRS pattern.^10,11^ Nevertheless, so far, little success has been achieved when trying to detect ‘invisible’ abnormalities of myocardial depolarization in standard clinical 10-s electrocardiograms (ECG).

We have recently reported a method for QRS complex analysis that might be used for this purpose.^12^ Briefly, the method is based on projecting the 12-lead ECG signal (i.e. its eight independent leads) into an optimized three-dimensional orthogonal representation, reconstructing the 12-lead signal back from the orthogonal leads, and measuring the difference between the original and the reconstructed signal. As also recently reported, the ECG reconstruction methods allow to differentiate between noise and signal components that might be attributed to localized heterogeneities of the depolarization wavefront.^12^ Only small proportions (single-digit percentages) of the original ECG signal are attributable to such localized heterogeneities but we propose that these measurements might be interpreted as invisible QRS ‘micro-fragmentation’ (QRS-*μf*).

We have tested the predictive value of QRS-*μf* in three independent populations with different risk of cardiac adverse events. We show here that in these tests, QRS-*μf* not only significantly predicted adverse outcome during follow-up but that the risk prediction was also independent of other recognized risk factors including the visually detected QRS fragmentation (that, for the distinction purposes, we call ‘macro-fragmentation’, QRS-*Mf*).

## Methods

### Investigated populations and electrocardiographic recordings

All three sources of analysed ECGs and follow-up data have previously been published.^13–15^

#### Retrospective part of EU-CERT-ICD

The European Commission supported study EU-CERT-ICD included a retrospective part that recorded patients in whom automatic implantable cardioverter-defibrillators (ICDs) were implanted for primary prophylaxis between 2002 and 2014. All details of this part of EU-CERT-ICD were reported previously.^13^ In five contributing centres (Basel, Göttingen, Leuven, Oulu, and Utrecht) short-term (8- or 10-s) digital 12-lead ECG recordings were also collected in the patients on median of 1 day [interquartile range (IQR) 1–6 days] before ICD implantation.

As previously described,^13^ clinical data in this registry included, among others, pre-implantation assessment of left ventricular ejection fraction (LVEF), rhythm classification of the recorded ECG, and the distinction between ischaemic and non-ischaemic heart disease.

#### VA Washington

A collection of digital 10-s 12-lead ECGs of US male veterans with ischaemic and non-ischaemic heart disease was made available for testing the predictive value of QRS-*μf*. As previously described,^14^ these were the historical data of patients recorded between 1984 and 1991 at the VA Medical Center in Washington, DC. All ECG recordings were stored within the hospital information system of the clinical centre. For the purposes of a previous study,^14^ least noise-polluted ECG recording was selected for each patient. These recordings were available for the present investigation.

#### Whitehall II study

The Whitehall II programme is an ongoing epidemiologic study with repeated calls during which series of medical investigations are performed in British civil servants of a broad spectrum of employment levels and positions.^15^ During the call between 2007 and 2009, participants in sinus rhythm had a digital 5-min 12-lead ECG recorded.^16^ To model short-term 10-s ECG acquisition, a 10-s section was extracted starting 100 s from the beginning of the 5-min signal.

### Electrocardiogram analyses

Supplementary material online, *Table S1* shows the technical details of the analysed ECGs. In each short-term ECG, the locations of QRS complexes were detected automatically and, where necessary, checked and corrected visually. Subsequently, the ECG signals were filtered (100 Hz low pass Butterworth filter with cubic spline baseline wander elimination—see Supplementary material online, *Figure S1* for details). Using these filtered signals, representative median beats were constructed in each lead and superimposed on the same isoelectric axis. All these pre-processing used algorithms and their software implementation that were repeatedly used and validated in previous studies.^12,17^

#### Electrocardiogram measurements

For each of the analysed ECGs, the image of the superimposed patterns of representative beats of different leads was visually interpreted and global 12-lead QRS onset and offset, and T wave offset points were identified. Supplementary material online, *Figure S1* also shows an example of measured ECG patterns. The visual analyses were made by team members who had neither access to any clinical and/or follow-up data nor information of a study from which individual ECGs originated. To assure consistency of the visual ECG interpretation, the recordings of each of the data sources were interpreted by the same team member.

In each ECG, heart rate was measured based on the total duration of the short-term recording. QT interval was measured from the global onset of the QRS complex to the global offset of the T wave, and spatial angle between the QRS complex and the T wave loops was measured by the previously published method^18,19^ of the total cosine R to T (TCRT) and expressed in degrees. To obtain QTc values, the QT interval was corrected for the heart rate using Fridericia formula.

Since the construction of representative median beat eliminates patterns not synchronized with QRS complexes, the interval measurements were also reliable in atrial fibrillation recordings that were present in the EU-CERT-ICD data—note Supplementary material online, *Figure S1*. (This was consistent with previously published analyses of ECGs polluted by other types of biological noise not phase-locked with QRS complexes.^17^) Similarly, correction of the QT interval to the heart rate derived from the complete 10-s recordings made QTc calculations also applicable to atrial fibrillation ECGs.

#### QRS macro-fragmentation

Using the filtered median beat images of the individual ECG leads, QRS-*Mf* was defined as additional QRS local maxima,^3–5^ i.e. as a visible notching in the pattern of the R or S waves, including additional waves, if present in more than one lead.

#### QRS micro-fragmentation

The term *micro-fragmentation* should not be interpreted as an increased precision of the ‘standard’ visible macro-fragmentation. Rather, we propose this term since the analysis detects signal characteristics that are largely inaccessible by the naked eye and which are, in principle, independent of the visually detected QRS-*Mf*.

**Figure 1:**
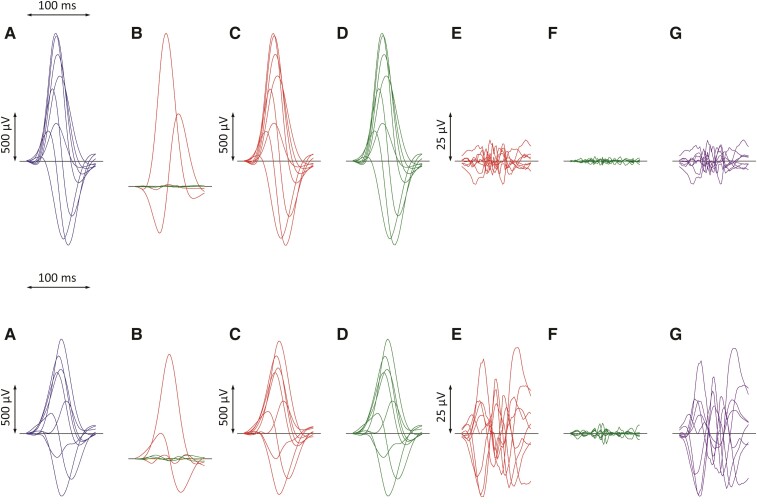
Example of ECG processing of a recordings in a 69-year-old male survivor (top row) and a 60-year-old patient who died 11 months later (bottom row). In both cases, the QRS duration was 109 ms. Filtered QRS complex patterns of independent Leads I, II, V1, V2, …, V6 are considered together as if on the same isoelectric axis (*A*). Singular value decomposition transforms the signals into eight algebraically orthogonal signals which are sorted according to their contribution to the original ECG leads (Components 1–3 are shown in red, 4–6 in green, and 7 and eight in amber in panels (*B*); the 7th and 8th components are almost invisible in these cases). The Components 1–3 create the optimized three-dimensional QRS vector projection. When these components are used to reconstruct the original ECG, patterns in panels (*C*) are obtained while reconstruction based on Components 1–6 gives patterns in panels (*D*). (*E*) and (*F*) show the differences between the original ECG are the reconstruction based on 1–3 and 1–6 components, respectively (i.e. *E* = *A*–*C*, *F* = *A*–*D*). The residuals shown in panels (*F*) (corresponding to the contribution of 7th and 8th components) are considered noise and eliminated. QRS micro-fractionation is calculated as the averaged absolute area under contribution by Components 4–6 shown in panels *G* (*G* = *D*–*C* = *E*–*F*). This area is related to the absolute area under the original ECG signal and was 0.887 and 5.754% in the top and bottom row ECGs, respectively. Note that the differences between panels (*A*) and (*C*) cannot be visually quantified.

Individual steps of the QRS-*μf* analysis are summarized in *Figure 1*: QRS-*μf* was quantified using the published method based on singular value decomposition (SVD) of the QRS complex signal (that is, between the visually detected and manually verified QRS onset and offset). Singular value decomposition considers the signals of all eight algebraically independent leads of 12-lead ECG (Leads I, II, V1, V2, …, V6) and examines the multi-lead signal in a theoretical eight-dimensional space. Within this representation, it composes eight algebraically orthogonal signals which are sorted according to their contribution to the original ECG. The 1st, 2nd, and 3rd components correspond to a three-dimensional representation of the ECG (as if the XYZ leads were rotated to contain the maximum signal in lead X, most of the signal perpendicular to X in Lead Y, and the reminder of the three-dimensional representation in Lead Z). When the ECG is reconstructed back from these rotated XYZ components, the reconstruction differs from the original ECG and the difference is represented by the 4th–8th components of the original SVD decomposition.

The numerical values of QRS-*μf* are the sums of 4th, 5th, and 6th decomposition fractions while the 7th and 8th fractions are attributed to recording noise. As previously explained, micro-fragmentation values are expressed in percentages of the total area under the absolute QRS complex curves and averaged over all eight independent leads of the analysed ECG.^12^ A certain level of micro-fragmentation is found within each ECG (i.e. each ECG differs from its optimized XYZ reconstruction) but the observed distribution of QRS-*μf* estimates among healthy subjects proposed that values above 3.5% might be considered abnormal.^12^

The same method for micro-fragmentation calculation (including the same ECG pre-processing) as previously published^12^ was used in all three studies. The QRS-*μf* estimates were based on the initial assessment of QRS onset and offset points that were not changed during the analysis. Since the analysis was based on the processing of median representative beats, the method was also applicable to atrial fibrillation recordings (note Supplementary material online, *Figure S1*).

### Follow-up events

For the purposes of this study, all-cause mortality data were available from EU-CERT-ICD and VA Washington studies. In the Whitehall II study, mortality data were available including the distinction between cardiovascular and non-cardiovascular deaths. This distinction was based on the cause of death in the death certificates (i.e. cardiovascular death was defined as death due to the ICD-10 coded diseases of the circulatory system).

Consequently, in the analyses of the predictive value of QRS-*μf* assessment, all-cause mortality was considered in the cardiac patients of EU-CERT-ICD and of VA Washington studies while cardiovascular death was considered in the general-population Whitehall II study (we have not aimed at predicting the substantial proportion of Whitehall II mortality due to neoplasms). In all these studies, follow-up from the date of the ECG acquisition was considered, restricted to a maximum of 5 years.

When using the terms survivors, non-survivors, and mortality in this text, we shall mean the follow-up distinction between those who did not and did die in the EU-CERT-ICD and VA Washington populations and those who did not and did die of cardiovascular death in the Whitehall II population.

### Statistics and data presentation

Categorical data are presented as percentages with absolute counts where appropriate, continuous data are shown as medians and IQR. In each of the populations, differences in continuous risk predictors between survivors and non-survivors were tested by non-parametric Mann–Whitney *U*-test with cumulative distributions of QRS-*μf* values displayed. Differences between cumulative distributions were further evaluated using Kolmogorov–Smirnov test which was also used to compare the QRS-*μf* distributions between subjects with and without QRS-*Mf* diagnosed. Relationships between different continuous risk factors were investigated using Kendall’s *τ* coefficients.

Associations between QRS-*μf* measurements and mortality during the follow-up were compared with other risk predictors of age, heart rate, QRS duration, QTc duration, TCRT, and the presence of QRS-*Mf*. In the EU-CERT-ICD study, comparison with LVEF was also included. Using univariable and multivariable Cox regression models with backward stepwise elimination, hazard ratios (HRs) and their 95% confidence intervals (CIs) of each risk factor were estimated for each of the source studies twice—with continuous and dichotomized risk predictors. In the models using continuous risk factors, numerical measurements of QRS-*μf* were used after logarithmic transformation. The dichotomized models also included the presence of QRS-*Mf*. The same dichotomies of the risk factors were used in all three source studies: dichotomy of 3.5% of QRS-*μf* was prospectively applied^12^; age was dichotomized at >65 years; heart rate at >75 b.p.m.; QRS duration at >120 ms; QTc duration at >450 ms and TCRT at >110°. In the analysis of EU-CERT-ICD study, LVEF was dichotomized at <25% since this value was close to the median of the population and, in supplementary analyses, creatinine level was dichotomized at 1.35 mg/dL. For QRS-*μf* and other continuous variables, Harrell’s *C*-index values were calculated^20^ together with areas under the receiver operating characteristic (ROC) curve and their Mann–Whitney standard errors. Receiver operating characteristic curves considered deaths during complete follow-up.

To investigate the additional predictive value of QRS-*μf* in comparison with QRS-*Mf*, the univariable predictive value of QRS-*μf* was assessed in sub-populations of subjects without diagnosed QRS-*Mf*.

Comparison of the probability of mortality in dichotomized populations was displayed using Kaplan–Meier curves that were compared by log-rank test. The statistical testing was performed using SPSS package version 27 (IBM, Armonk, NY, USA). *P*-values < 0.05 were considered statistically significant; all tests were two-sided, no test multiplicity correction was used.

The EU-CERT-ICD study was also used to test the stability of the mortality risk prediction in different subsets of the data, by repeating the analyses in the data of different clinical centres, and in the data of patients diagnosed with ischaemic vs. non-ischaemic heart disease. In supplementary analyses, the predictive value of QRS-*μf* was also assessed among atrial fibrillation patients. Survival of subjects with QRS-*μf* above and below 3.5% was also compared in different population subgroups.

## Results

Table 1 shows the characteristics of the investigated populations. Taking these together, QRS-*μf* was investigated in 7779 subjects of whom 504 have not survived during the first 5 years of follow-up. Nevertheless, mortality was very different between the populations. In the ICD-protected cardiac patients of EU-CERT-ICD, the cumulative 5-year death rate was 15.1%, while it was 21.3% in the cardiac VA Washington patients. In the general population of the Whitehall II study, the 5-year rate of cardiovascular mortality was 0.95% (while that of non-cardiovascular mortality, mainly due to neoplasms, was 2.3%). The prevalence of QRS-*μf* > 3.5% among the EU-CERT-ICD, VA Washington, and Whitehall II subjects was 45.7, 23.3, and 10.9%, respectively.

**Table 1:** Characteristics of the investigated populations

	Survivors	Non-survivors	*P*-value
EU-CERT-ICD			
*N*	1654	294	
Age (years)	64 (55–71)	69 (62–75)	<0.001
Females/males	336/1318	45/249	0.046
Heart rate (b.p.m.)	68.9 (59.8–79.2)	72.9 (65.0–83.9)	<0.001
LVEF (%)	26 (21–31)	25 (20–30)	<0.001
QRS duration (ms)	128 (112–159)	147 (122–171)	<0.001
QTc (ms)	442 (419–467)	452 (428–485)	0.001
TCRT (°)	151.0 (115.5–165.1)	159.9 (142.7–167.4)	<0.001
QRS micro-fragmentation (%)	3.213 (2.386–4.548)	4.188 (3.005–5.598)	<0.001
VA Washington			
*N*	598	162	
Age (years)	62 (56–68)	65 (59–69)	<0.001
Heart rate (b.p.m.)	72 (61–85)	79 (66–91)	<0.001
QRS duration (ms)	110 (103–119)	112 (104–128)	0.010
QTc (ms)	419 (404–435)	420 (406–449)	0.029
TCRT (°)	102.7 (69.3–140.2)	120.6 (79.9–155)	0.003
QRS micro-fragmentation (%)	2.143 (1.586–3.155)	2.521 (1.736–4.104)	0.001
Whitehall II^a^			
*N*	5023	48	
Age (years)	65 (61–70)	70 (61–74)	0.001
Females/males	1360/3663	8/40	0.140
Heart rate (b.p.m.)	67 (60–74)	72 (59–78)	0.102
QRS duration (ms)	106 (100–111)	107 (103–114)	0.068
QTc (ms)	423 (412–436)	426 (414–444)	0.126
TCRT (°)	45.3 (28.3–76.3)	74.4 (38.3–132.8)	<0.001
QRS micro-fragmentation (%)	1.944 (1.437–2.645)	2.439 (1.807–3.581)	0.001

The table shows medians and interquartile ranges and their comparison between 5-year survivors and non-survivors. TCRT = total cosine R to T.

a For the Whitehall II study, comparison is shown between those who did not and did die of cardiovascular death during a 5-year follow-up. Non-parametric Mann–Whitney *P*-values are shown for the comparison of numerical factors between survivors and non-survivors. The *P*-values of the differences between the proportions of non-survivors among females and males were obtained by Fisher exact test. *P*-values (log-rank test) of the differences of female vs. male Kaplan–Meier survival curves were 0.110 and 0.106, in the EU-CERT-ICD and Whitehall II populations, respectively. (Note that the VA Washington population included only males.)

Supplementary material online, *Table S2* shows that in all three populations, the relationship between QRS-*μf* and other risk factors considered in the analysis was very weak although it was, not surprisingly, frequently statistically significant because of the large sample sizes. In all populations, the relation of QRS-*μf* to QRS duration was stronger than to other risk factors but this was still weak with the correlation *τ* coefficients well below 0.4. Since QRS-*μf* is calculated as a proportion to the total area under the QRS waves, the relation to the QRS complex duration is not caused by its mathematical definition.^12^

### Outcome prediction

**Figure 2:**
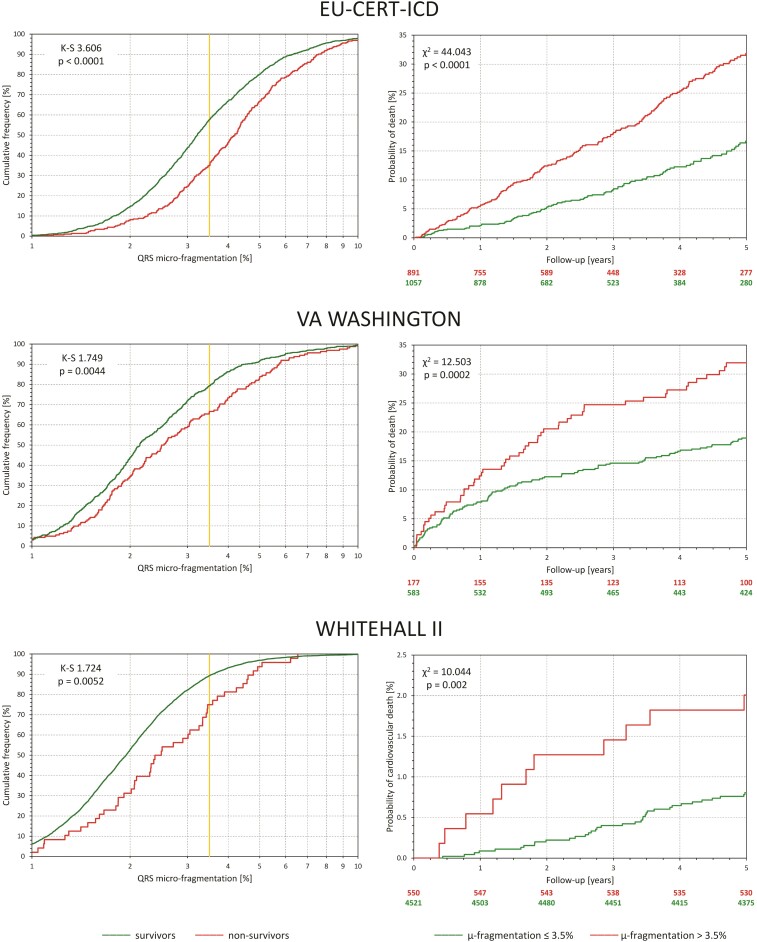
For each of the investigated populations, the left panel shows the comparison between distributions of QRS micro-fragmentation values in survivors (green line) and non-survivors (red line). The distributions were compared by Kolmogorov–Smirnov test (K–S statistics values shown). The yellow vertical lines mark the 3.5% dichotomy. The right panels show the Kaplan–Meier probabilities of non-survival for subjects with QRS micro-fragmentation ≤3.5% (green line) and >3.5% (red line). Numbers of subjects at risk are shown below the panel in corresponding colours. The non-survival probabilities were compared by log-rank test.

The left panels of individual sections of *Figure 2* show, for each of the investigated populations, cumulative distributions of QRS-*μf* in survivors and non-survivors. In all three cases, the distributions were highly statistically different. The right panels of the figure show the survival probabilities in sub-populations with QRS-*μf* > 3.5 and ≤3.5%. In all three populations, the differences were highly statistically significant.

*Tables 2* and *3* show the results of the Cox regression models using continuous and dichotomized risk predictors. In both models, QRS-*μf* was found to be an independent highly significant outcome predictor in all three populations. Harrell’s C-index values and univariable areas under the ROC curves of continuous risk predictors are shown in Supplementary material online, *Figure S2*. Supplementary material online, *Table S3* shows multivariable C-index statistics. Selected multivariable ROC curves are shown in Supplementary material online, *Figure S3*.

**Table 2:** Association between mortality^a^ and continuous values of risk factors

	Univariable analysis			Multivariable analysis^b^		
	Wald	*P*-value	HR (95% CI)	Wald	*P*-value	HR (95% CI)
EU-CERT-ICD						
Age (years)	48.9	<0.001	1.043 (1.031–1.055)	30.9	<0.001	1.035 (1.022–1.047)
Heart rate (b.p.m.)	30.8	<0.001	1.019 (1.012–1.026)	24.2	<0.001	1.018 (1.011–1.025)
LVEF (%)	30.1	<0.001	0.959 (0.945–0.973)	9.17	0.002	0.975 (0.958–0.991)
QRS duration (ms)	26.6	<0.001	1.009 (1.006–1.013)			
QTc (ms)	20.3	<0.001	1.006 (1.004–1.009)			
TCRT (°)	26.1	<0.001	1.010 (1.006–1.014)	7.39	0.007	1.006 (1.002–1.010)
log_2_ (QRS micro-fragmentation)	42.9	<0.001	1.688 (1.443–1.975)	25.5	<0.001	1.540 (1.302–1.821)
VA Washington						
Age (years)	12.4	<0.001	1.029 (1.013–1.046)	11.4	0.001	1.029 (1.012–1.047)
Heart rate (b.p.m.)	13.6	<0.001	1.014 (1.007–1.022)	10.2	0.001	1.013 (1.005–1.021)
QRS duration (ms)	12.1	0.001	1.005 (1.002–1.008)			
QTc (ms)	7.25	0.007	1.007 (1.002–1.013)			
TCRT (°)	9.71	0.002	1.006 (1.002–1.009)	4.29	0.038	1.004 (1.000–1.007)
log_2_ (QRS micro-fragmentation)	13.3	<0.001	1.422 (1.176–1.719)	10.1	0.002	1.367 (1.127–1.659)
Whitehall II						
Age (years)	11.6	0.001	1.088 (1.036–1.142)	7.79	0.005	1.072 (1.021–1.126)
Heart rate (b.p.m.)	5.41	0.020	1.026 (1.004–1.049)			
QRS duration (ms)	6.50	0.011	1.021 (1.005–1.037)			
QTc (ms)	0.52	0.472	0.997 (0.987–1.006)			
TCRT (°)	24.4	<0.001	1.016 (1.009–1.022)	15.5	<0.001	1.013 (1.006–1.019)
log_2_ (QRS micro-fragmentation)	12.2	<0.001	1.972 (1.347–2.887)	5.12	0.024	1.555 (1.061–2.279)

CI, confidence interval; HR, hazard ratio; LVEF, left ventricular ejection fraction; TCRT, total cosine R to T.

a The outcome is all-cause mortality for the EU-CER-ICD and VA Washington studies and cardiovascular mortality for the Whitehall II study.

b Multivariable analysis used backwards stepwise elimination. In addition to hazard ratios, Wald statistics are shown. QRS micro-fragmentation was used after logarithmic transformation with base 2—hazard ratios correspond to value increases by a factor of 2.

**Table 3:** Association between mortality^a^ and dichotomized risk factors

	Prevalence (%)	Univariable analysis			Multivariable analysis^b^		
		Wald	*P*-value	HR (95% CI)	Wald	*P*-value	HR (95% CI)
EU-CERT-ICD							
Age > 65 years	49.6	43.1	<0.001	2.269 (1.777–2.897)	34. 1	<0.001	2.097 (1.635–2.688)
Female sex	19.6	0.51	0.476	0.891 (0.648–1.224)			
Heart rate > 75 b.p.m.	35.3	19.7	<0.001	1.685 (1.338–2.121)	20.6	<0.001	1.713 (1.358–2.161)
LVEF < 25%	37.5	14.5	<0.001	1.563 (1.241–1.967)	9.68	0.002	1.444 (1.146–1.821)
QRS duration > 120 ms	62.3	24.9	<0.001	2.010 (1.528–2.643)			
QRS macro-fragmentation	32.3	9.59	0.002	1.443 (1.144–1.820)			
QTc > 450 ms	42.3	8.05	0.005	1.393 (1.108–1.752)			
TCRT > 110°	41.3	14.2	<0.001	1.554 (1.235–1.954)			
QRS micro-fragmentation >3.5%	45.7	41.8	<0.001	2.208 (1.737–2.808)	30.1	<0.001	1.987 (1.555–2.540)
VA Washington							
Age > 65 years	36.7	8.27	0.004	1.573 (1.156–2.142)	6.32	0.012	1.542 (1.100–2.163)
Heart rate > 75 b.p.m.	45.2	14.7	<0.001	1.846 (1.350–2.526)	8.03	0.005	1.645 (1.166–2.322)
QRS duration > 120 ms	24.1	8.81	0.003	1.644 (1.184–2.283)			
QRS macro-fragmentation	15.2	9.48	0.002	1.779 (1.233–2.568)			
QTc > 450 ms	13.8	11.7	0.001	2.011 (1.347–3.002)	6.49	0.011	1.711 (1.132–2.586)
TCRT > 110°	54.1	5.31	0.021	1.455 (1.058–2.002)	3.93	0.048	1.432 (1.004–2.044)
QRS micro-fragmentation >3.5%	23.3	12.2	<0.001	1.789 (1.290–2.480)	5.86	0.015	1.578 (1.091–2.283)
Whitehall II							
Age > 65 years	47.5	6.86	0.009	2.230 (1.224–4.065)	5.06	0.025	1.997 (1.093–3.647)
Female sex	27.0	2.54	0.111	0.540 (0.253–1.153)			
Heart rate > 75 b.p.m.	22.8	7.46	0.006	2.239 (1.255–3.993)	7.26	0.007	2.214 (1.241–3.949)
QRS duration > 120 ms	6.4	7.96	0.005	2.983 (1.396–6.373)			
QRS macro-fragmentation	6.4	7.83	0.005	2.955 (1.383–6.312)			
QTc > 450 ms	8.0	1.33	0.249	1.654 (0.703–3.891)			
TCRT > 110°	14.6	18.0	<0.001	3.548 (1.978–6.365)	13.8	<0.001	3.073 (1.698–5.564)
QRS micro-fragmentation >3.5%	10.8	9.23	0.002	2.753 (1.432–5.290)	5.51	0.019	2.214 (1.140–4.300)

CI, confidence interval; HR, hazard ratio; LVEF, left ventricular ejection fraction; TCRT, total cosine R to T.

a The outcome is all-cause mortality for the EU-CERT-ICD and VA Washington studies and cardiovascular mortality for the Whitehall II study.

b Multivariable analysis used backwards stepwise elimination. In addition to hazard ratios, Wald statistics are shown. Note that the VA Washington population included only males.

#### Stability of outcome prediction

**Figure 3:**
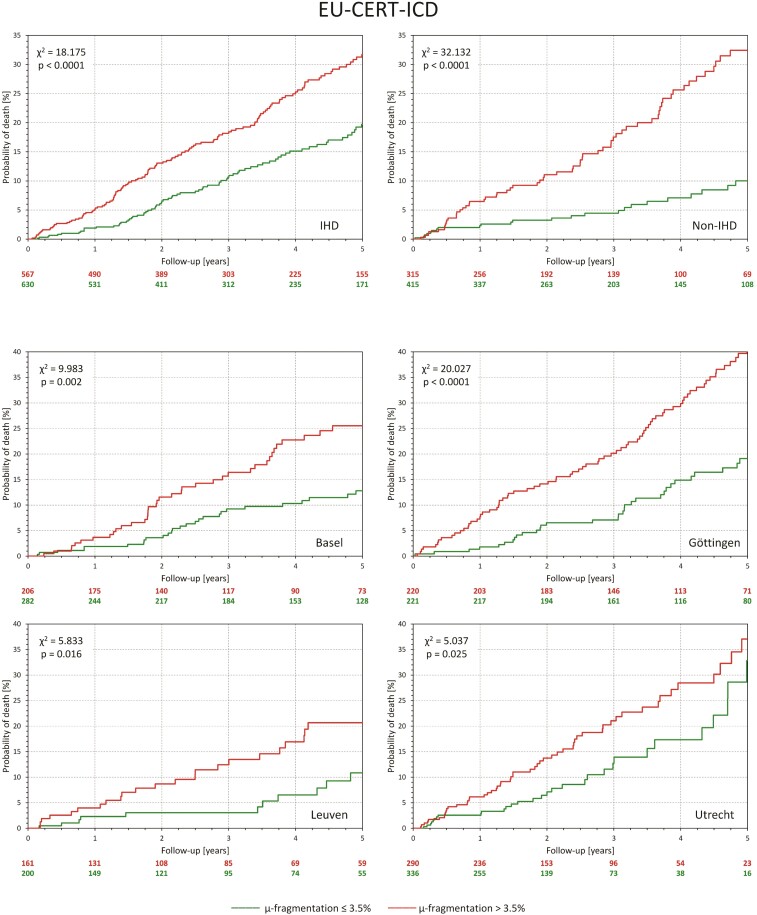
All panels show Kaplan–Meier probabilities of death in different subgroups of the EU-CERT-ICD population. Green and red lines correspond to patients with QRS micro-fragmentation ≤3.5 and >3.5%, respectively. The top two panels show sub-populations with ischaemic heart disease and non-ischaemic heart disease. The bottom four panels correspond to the sub-populations of four different centres that contributed more than 100 patients. Numbers of patients at risk are shown below each panel in corresponding colours. The non-survival probabilities were compared by log-rank test.

The top part of *Figure 3* shows that when the distinction between QRS-*μf* > 3.5 and ≤3.5% was applied separately to ischaemic and non-ischaemic patients of EU-CERT-ICD, highly significant differences in the survival probability were seen in both groups. Supplementary material online, *Table S4* shows that in both groups QRS-*μf* was a significant mortality predictor also in multivariable Cox regression models.

Of the 1948 patients of EU-CERT-ICD, clinical centres in Basel, Göttingen, Leuven, Oulu, and Utrecht contributed ECGs of 488, 441, 361, 32, and 626 patients, respectively (see Supplementary material online, *Table S5*). Stability of survival probability prediction was therefore tested in separate per-centre data of Basel, Göttingen, Leuven, and Utrecht. Kaplan–Meier probabilities of death distinguishing QRS-*μf* >3.5 and ≤3.5% are shown in the bottom part of *Figure 3*. In all four cases, the distinction was statistically significant. Supplementary material online, *Table S6* shows that in multivariable Cox regression analysis, QRS-*μf* was found to be an independent predictor of survival in the data of three of the four centres.

#### Outcome prediction in clinical subgroups

Supplementary material online, *Figures S4–S6* show that comparisons of death probabilities between QRS-*μf* >3.5 and ≤3.5% sub-strata of different EU-CERT-ICD well-defined subgroups were all statistically significant. The same outcome differences were seen in VA Washington and Whitehall II sub-populations, although statistical significance was not always reached because of small group sizes.

Supplementary material online, *Figure S7* shows that comparisons of death probabilities between QRS-*μf* >3.5 and ≤3.5% were also statistically significant in EU-CERT-ICD patients with pre-implantation creatinine levels above and below 1.35 mg/dL, as well as in patients who, for clinical reasons, had and had not a defibrillator implanted with cardiac resynchronization function.

Finally, Supplementary material online, *Figure S8* shows that among the EU-CERT-ICD patients, the statistical significance of death prediction by QRS-*μf* >3.5% was not influenced by clinical decisions leading to intention to treat with beta-blockers, amiodarone, and statins.

#### Outcome prediction in atrial fibrillation patients

Among the EU-CERT-ICD patients, 214 (11.0%) suffered from atrial fibrillation. The 5-year survival of these patients (76.6%) was substantially worse than that of patients with sinus rhythm recordings (87.4%, *P* < 0.001). *Structured Graphical Abstract* and Supplementary material online, *Figure S7* shows that QRS-*μf* >3.5 and ≤3.5% provided significant survival separation also in atrial fibrillation patients. In a multivariable analysis, QRS-*μf* was the by far strongest mortality predictor in atrial fibrillation patients (see Supplementary material online, *Table S7*). Similarly, when using Cox regression analysis of dichotomized risk values, QRS-*μf* >3.5% was the only statistically significant risk predictor surviving the multivariable analysis in the atrial fibrillation subgroup (with a HR of 1.972, 95% CI of 1.101–3.534, *P* = 0.022).

### Comparison of macro- and micro-fragmentation

**Figure 4:**
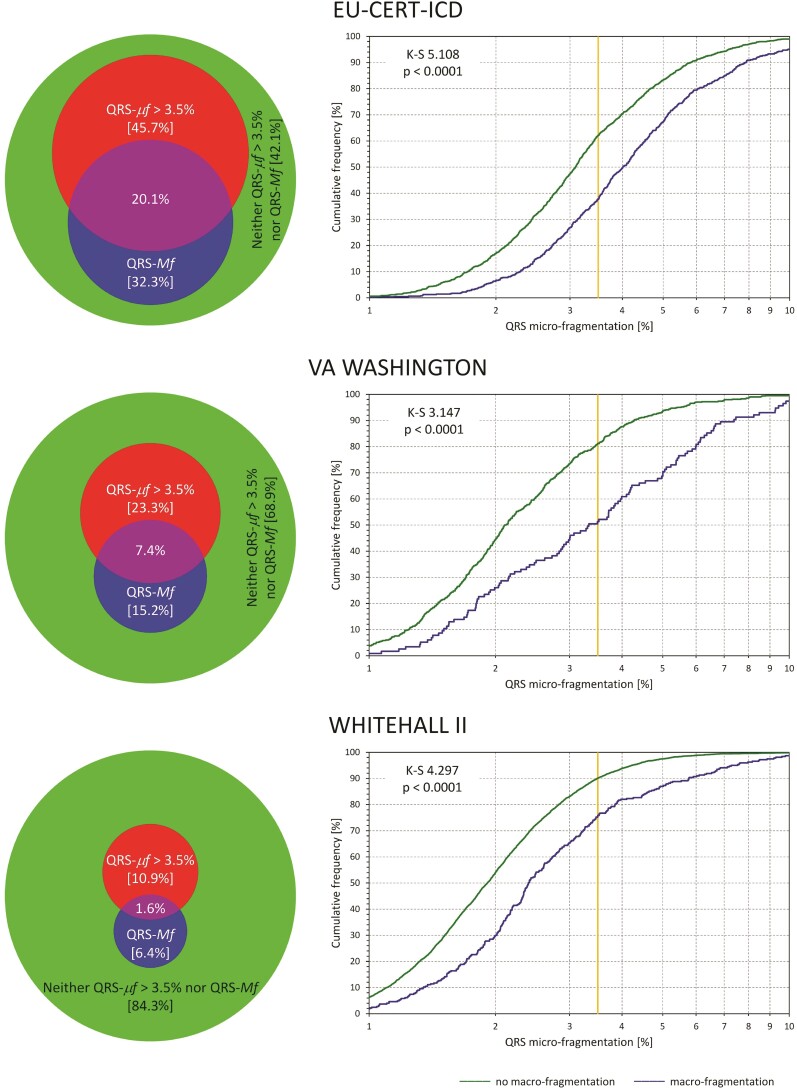
For each of the investigated populations, the scaled Venn diagram on the left shows the proportions of subjects observed. Red circle: QRS micro-fragmentation >3.5% (QRS-*μf* >3.5%). Blue circle: QRS macro-fragmentation (QRS-*Mf*). Violet overlap between the red and blue circle: Both QRS macro-fragmentation and QRS micro-fragmentation >3.5%. Green reminder of the background circle: No QRS macro-fragmentation and QRS micro-fragmentation ≤3.5%. The sizes of the red and blue circles are in proportion of the background circle corresponding to the total population. The percentages of the categories are shown. The panels on the right show the comparisons between distributions of QRS micro-fragmentation values in subjects with (blue line) and without observed QRS macro-fragmentation (green line). The distributions were compared by Kolmogorov–Smirnov test (K–S statistics values shown). The yellow vertical lines mark the 3.5% dichotomy.

**Figure 5:**
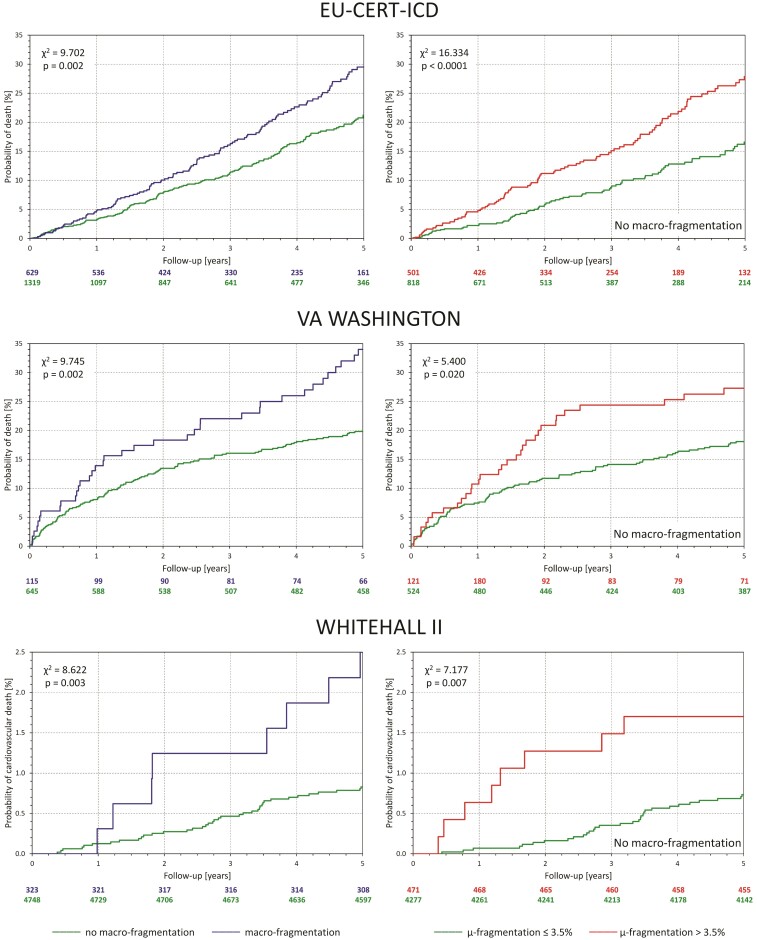
For each of the investigated populations, the panel on the left shows Kaplan–Meier probabilities of non-survival for subjects with (blue line) and without observed QRS macro-fragmentation (green line). The panels on the right show Kaplan–Meier probabilities of non-survival in subjects without QRS macro-fragmentation stratified by QRS micro-fragmentation above (red line) and below (green line) of the 3.5% dichotomy. Numbers of patients at risk are shown below each panel in corresponding colours. The non-survival probabilities were compared by log-rank test.

The left panels of *Figure 4* show, for the separate investigated populations, the distribution of cases of positive QRS-*μf* and QRS-*Mf* (when the positive QRS-*μf* was defined >3.5%). The scaled Venn diagrams show that positive QRS-*μf* and QRS-*Mf* overlapped but were far from identical. The right panels of *Figure 4* show the distributions of QRS-*μf* values among subjects with and without observed QRS-*Mf*. In all three populations, the QRS-*μf* values were significantly larger in subjects with QRS-*Mf*. The left panels of *Figure 5* show the comparisons of mortality between patients with and without observed QRS-*Mf*. In all three populations, the differences were statistically significant albeit somewhat less strong compared with the survival differences stratified by QRS-*μf* as shown in *Figure 2*. The right panels of *Figure 5* show that when subjects with observed QRS-*Mf* are excluded, QRS-*μf* dichotomized at 3.5% still significantly separated high- and low-risk subjects in all three populations.

Supplementary material online, *Figure S9* shows that when only subjects with QRS-*μf* ≤3.5% were considered, the presence or absence of QRS-*Mf* did not lead to statistically significant survival differences in EU-CERT-ICD or in VA Washington data. It did lead to a significant survival difference in the Whitehall II data albeit less strongly significant compared with the opposite combination of QRS-*Mf* and QRS-*μf*.

## Discussion

The study shows convincingly that the newly described ECG analysis that quantifies QRS-*μf* provides a powerful mortality predictor independent of other established risk factors. We observed this in populations with different clinical characteristics and different risk profiles. Equally importantly, we observed this also in clinically well-defined sub-populations. In the EU-CERT-ICD data, this included sub-populations of patients with ischaemic and non-ischaemic heart disease as well as atrial fibrillation patients (*Structured Graphical Abstract* and Supplementary material online, *Figure S7* and *Table S7*). This is of additional importance since a number of previously proposed ECG-based risk stratifiers^21,22^ are not applicable to atrial fibrillation recordings.

This strong risk predictor is based on a standard 10-s 12-lead ECG, i.e. on a clinical test that is routinely and repeatedly performed in the vast majority of healthcare settings. It thus might be widely applied.

### Outcome prediction

We show the association of QRS-*μf* with mortality risk compellingly. Because of the data character, QRS-*μf* relates to cardiovascular mortality. In the EU-CERT-ICD and VA Washington populations of severe cardiac patients, all-cause deaths were reasonable approximations of cardiovascular mortality. Contrary to cardiovascular mortality, non-cardiovascular mortality was not significantly predicted in the Whitehall II data (details not shown).

Since the very strong distinction between low- and high-risk strata was observed among the EU-CERT-ICD patients who were all ICD protected, it seems more likely that increased QRS-*μf* signifies tendency to heart failure rather than propensity to arrhythmic complications. This also appears to agree with previous observations that linked QRS-*Mf* to myocardial scarring, interstitial fibrosis, and subclinical myocardial damage.^23,24^ We hypothesize that increased QRS-*μf* reflects similar abnormalities. This is further supported by our observation (details not shown) that when QRS-*μf* was used to predict first appropriate ICD shock rather than death in the EU-CERT-ICD data, the univariable prediction was only borderline significant (*P* = 0.044, log_2_QRS-*μf* HR of 1.216 with 95% CI of 1.005–1.472). Although we do not have data on anti-tachycardia pacing therapy (and might have thus missed some sustained tachycardia episodes that the ICDs terminated without using a shock), we believe that the prediction of ICD shocks would have been stronger if increased QRS-*μf* were not predominantly linked to non-arrhythmic complications. Indeed, in agreement with our observation of strong QRS-*μf*-based risk prediction among non-ischaemic ICD patients, abnormalities within signal-averaged QRS complex were previously observed among hypertrophic cardiomyopathy patients who died non-suddenly (but not in those who died suddenly).^25^

The differences between the EU-CERT-ICD and Washington VA populations deserve a more detailed explanation. While the 5-year mortality was greater in the Washington VA population (21.3%) than in the EU-CERT-ICD population (15.1%), the QRS-*μf* measurements led to larger values in the EU-CERT-ICD recordings (*Table 1*). This suggests that lower mortality rates in EU-CERT-ICD existed despite the observation of more pronounced ECG depolarization heterogeneity. This is explained not only by the defibrillator protection of the EU-CERT-ICD patients but also by the time span of some two decades between these two data collections. More recent advances of clinical care thus necessarily reduced the EU-CERT-ICD mortality.

### Micro- and macro-fragmentation

As previously explained, QRS-*μf* computation considers all eight independent ECG leads together and extracts as much as possible of the multi-lead signal that could be attributed to the movement of a depolarization dipole in three orthogonal dimensions. The rest of the eight-lead signal is explained by movements in ‘algebraic’ 4th, 5th and so on dimensions always taking as much as possible of the remaining signal into the next dimension. This means that QRS-*μf* (sum of the components in the 4th to 6th dimensions) represents localized heterogeneities and abnormalities of the depolarization wavefront that cannot be explained by simple three-dimensional convolution of the depolarization dipole movement (otherwise, they would fit into the first three reconstructed dimensions) but still have a common expression in more than one or two ECG leads. Only the decomposition in the 7th and 8th dimensions is, based on the previous considerations, attributed to noise and signal imperfection.^12^

This consideration also explains the principal difference between QRS-*Mf* and QRS-*μf*. As already stated, micro-fractionation is not the refinement of visible macro-fractionation. Abnormal three-dimensional depolarization dipole movements might easily lead to dual R or S waves or other macro-fragmentation patterns while contributing little to the ECG signal beyond the three orthogonal dimensions (see Supplementary material online, *Figure S10*). On the contrary, even large values of QRS-*μf* might be associated with QRS complex patterns that are not visibly fragmented. Hence, QRS-*μf* should not be understood as increased precision of QRS-*Mf*. Both concepts complement but do not refine each other. In other words, QRS-*Mf* and QRS-*μf* do not need to correlate. Nevertheless, our findings indicate that when QRS-*Mf* is found without abnormally increase values of QRS-*μf*, the risk prediction is absent or substantially reduced.

In this sense, *Figures 4* and *5* must not be overinterpreted—although 3.5% dichotomy was prospectively applied, the continuous scale of QRS-*μf* makes the relationship to QRS-*Mf* more complex. Still, both QRS-*Mf and* QRS-*μf* depict abnormalities in the depolarization sequence. The Cox regression comparisons (*Table 3*) might suggest that the abnormalities that contribute to QRS-*μf* (also present in macro-fragmented QRS complexes) might be the mechanistic link between QRS abnormalities and worsened outcome. Nevertheless, more detailed analyses of other data are needed to elucidate this concept further.

### Covariates

Although the relationship between QRS-*μf* of QRS duration was rather weak (intentionally, we used Kendall’s *τ* to express the similarities of selecting high-risk population subgroups), such a relationship exists and was observed not only in these data but also in the previous analysis of ECGs of healthy subjects.^12^ The same considerations of the underlying component might therefore be also made for the risk prediction by increased QRS duration. Importantly, QRS duration (considered as a continuous variable or dichotomized at 120 ms) and diagnosis of QRS-*Mf* were both eliminated in the multivariable Cox regression models when QRS-*μf* was included.

Previous analyses aiming at the detection of conduction abnormalities hidden in the QRS complex were based on different analyses of high-fidelity signal-averaged ECG recordings, well beyond the clinical practicality of standard short-term ECGs that we have analysed. Their prediction strength was also modest.^11,26^ Analyses of quadrupolar ECG components were mainly applied to body surface maps and little is known on their predictive strength.^27^ While we have presently applied this type of SVD decomposition to QRS complex signals, the same analysis might also prove valuable for the T wave analysis (to quantify repolarization abnormalities with possible arrhythmic risk implications) and perhaps also P waves (to assess atrial electrophysiology abnormalities with possible links to atrial fibrillation risk).

### Practical implications

Evaluation of QRS-*μf* does not demand any advanced ECG interpretation. While the visual diagnosis of QRS-*Mf* might be disputable in borderline cases, QRS-*μf* assessment requires only defining the window between QRS onset and offset. Previous observations in healthy subjects suggest that no particular precision of this analysis window is needed^12^ and our preliminary observations in the EU-CERT-ICD data suggest that the same might also apply to clinical recordings in cardiac patients (details not shown) although we cannot comment on recordings in different clinical settings. The algorithms to assess QRS-*μf* are also not computationally demanding and could easily be linked to or implemented within the standard equipment for digital ECG acquisition. Expressing QRS-*μf* on a continuous scale also avoids the problem of categorical yes/no classification needed for QRS-*Mf* which might potentially be problematic in borderline cases.^28^

To demonstrate the predictive power of QRS-*μf*, the Cox regression models evaluated different risk factors as if in competition for a best predictor position. In practice, however, combinations of different risk indicators need to be considered. This particularly applies to factors that might be derived from a standard 12-lead ECG recording, e.g. the spatial QRS-T angle that we found, in agreement with previous observations,^18,19^ to be another independent risk indicator. Importantly, we found QRS-*μf* unrelated to other risk factors suggesting that in future studies, it is suitable to be combined with other ECG-based predictors (elementary example in Supplementary material online, *Figure S11*).

### Outlook

The assessment of QRS-*μf* appears ready to be implemented in ECG screening programmes and studies of patients at cardiac risk, especially if QRS-*μf* assessment is used as an initial risk indicator and followed by characteristics derived from biochemistry, long-term and high-fidelity ECGs, blood pressure, stress testing, cardiac imaging, etc. The HRs associated with an increased QRS-*μf* (similar to the Harrell’s C-index values and areas under the ROC curves) might be perceived as only modest although they compare favourably with other risk factors derived from the same short-term ECGs. To further utilize risk assessment based on QRS-*μf*, the value to predict different mortality modes (e.g. arrhythmic vs. heart failure) needs to be investigated. These distinctions were not available in the data that we analysed. Prospective ICD studies (including the prospective EU-CERT-ICD part^29^) could be helpful in that respect when sufficient follow-up data are available. If confirmed that increased QRS-*μf* implies a risk of ICD-non-preventable cardiac death, applications might include the selection of ICD candidates with low QRS-*μf* values (combined with other indicators^21,22^) to increase the device efficacy. Our observation that QRS-*μf*-based risk prediction was particularly strong in non-ischaemic patients (*Figure 3*) might importantly help stratifying these ICD candidates in whom the therapy is presently uncertain.^30^ Even without mechanistic details, future screening programmes and studies of haemodialysis, hypertensive, and diabetic patients would likely benefit from QRS-*μf* assessment since it might, especially if combined with other ECG-based factors, differentiate between patients who do and do not require enhanced clinical attention. QRS-*μf* might also be valuable in the assessment of cardiac resynchronization therapy since more synchronous myocardial activation should lead to a less convoluted three-dimensional depolarization front. Finally, since QRS-*μf* is designed to quantify any departures from regular myocardial activation wavefront, we also hypothesize that it might be helpful in early diagnosis of interstitial myocardial pathologies ranging from amyloidosis, sarcoidosis, and lipomatosis to cellular transplant rejection.

### Limitations

In the categorical analyses, we have only used the QRS-*μf* dichotomy at 3.5% as previously proposed based on independent data in healthy subjects.^12^ It is possible if not likely that this QRS-*μf* cut-off could be further optimized in the analysed populations. We have not attempted such optimizations since the prospective nature of the positive QRS-*μf* distinction would have been lost. Intentionally, we have compared QRS-*μf* mainly to risk factors that can be obtained from standard ECG recordings. In real-life situations, other risk indicators also need to be considered. These were, however, not available for the analysed populations and we thus could not have included other factors in the multivariable analyses. Similarly, future studies are needed to assess QRS-*μf*-based predictions of different mortality modes. We were unable to verify the predictive value in atrial fibrillation patients in the Washington VA and Whitehall II populations since by design of these data collections, only ECG obtained in sinus rhythm were available. Finally, both QRS-*μf* and QRS-*Mf*, similar to QRS width and QT interval duration, were assessed in representative median beats of filtered ECG signals. Since we used the filtering technique available from many previous ECG studies,^17^ optimizing ECG pre-processing and filter setting might further increase the predictive power of QRS-*μf* assessment.

## Conclusion

The presented analyses confirm that QRS-*μf* is a new potent risk indicator available from objective analysis of standard 12-lead ECGs. In three populations of different clinical characteristics and in a number of clinically defined sub-populations, including atrial fibrillation patients, we found this risk factor to be a predictor of mortality independent of several other previously established risk indices. It seems plausible to speculate that the electrophysiologic abnormalities that contribute to increased QRS-*μf* values are responsible for the predictive power of visible QRS fragmentation and perhaps also contributing to the predictive value of prolonged QRS complex.

## Supplementary Material

ehac085_Supplementary_Data

## Data Availability

The data underlying this article will be shared on reasonable request to the corresponding author.
