# Supplementary material for: QRS micro-fragmentation as a mortality predictor
Source: Eur Heart J. 2022 Feb 21;43(40):4177–91. doi: 10.1093/eurheartj/ehac085 (PMC9584751; doi:10.1093/eurheartj/ehac085)
Supplement: ehac085_Supplementary_Data [file ehac085_supplementary_data.pdf]

QRS micro-fragmentation as a mortality predictor

by

Katerina Hnatkova[1], Irena Andršová[2,3], Tomáš Novotný[2,3], Annie Britton[4], Martin Shipley[4],
Bert Vandenberk[5], David J Sprenkeler[6], Juhani Junttila[7], Tobias Reichlin[8], Simon Schlögl[9,10],
Marc A Vos[6], Tim Friede[11,10], Axel Bauer[12], Heikki V Huikuri[7], Rik Willems[5], Georg Schmidt[13,14],
Michael R Franz[15], Christian Sticherling[16], Markus Zabel[9,10], Marek Malik[1,3]

[1] National Heart and Lung Institute, Imperial College, London, England,
[2] Department of Internal Medicine and Cardiology, University Hospital Brno, Brno, Czech Republic,
[3] Department of Internal Medicine and Cardiology, Masaryk University, Brno, Czech Republic,
[4] Research Department of Epidemiology and Public Health, University College London, England,
[5] Department of Cardiovascular Sciences, University of Leuven, Leuven, Belgium,
[6] Department of Medical Physiology, University Medical Center Utrecht, Utrecht, The Netherlands,
[7] University Central Hospital of Oulu and University of Oulu, Finland,
[8] Department of Cardiology, Inselspital, Bern University Hospital, Bern, Switzerland
[9] Department of Cardiology and Pneumology, University Medical Center, Göttingen, Germany,
[10] German Center for Cardiovascular Research (DZHK), partner site Göttingen, Göttingen, Germany
[11] Department of Medical Statistics, University Medical Center Göttingen, Göttingen, Germany
[12] University Hospital for Internal Medicine III, Medical University Innsbruck, Innsbruck, Austria,
[13] Klinikum rechts der Isar, Technical University of Munich, Munich, Germany,
[14] German Center for Cardiovascular Research partner site Munich Heart Alliance, Munich, Germany,
[15] Veteran Affairs and Georgetown University Medical Centers, Washington, DC, USA,
[16] Department of Cardiology, University Hospital of Basel, Basel, Switzerland.

Supplementary Tables and Figures

Supplementary Tables and Figures are presented in the order
as they are referred to in the text of the article.

Supplementary Table 1

Recording characteristics of analysed short-term ECGs

Data source	Equipment model	Exported sampling frequency	Exported LSB resolution	Analysed ECG Duration
**EU-CERT-ICD**				
Basel	Schiller CS-200	500 Hz	4.0 µV	10 s
Göttingen	Schiller CS-200	500 Hz	4.0 µV	10 s
Leuven	GE MAC 5500	250 Hz	4.88 µV	10 s
Oulu	Mortara ELI 380	1000 Hz	2.5 µV	8 s
Utrecht	GE MAC 5500	500 Hz	4.88 µV	10 s
**VA Washington**				
VA Washington	Marquette MAC/MUSE	250 Hz	4.88 µV	10 s
**Whitehall II**				
Whitehall II	Getemed CM 3000-12 BT	1024 Hz	2.93 µV	10 s

Standard settings of the equipment were used with removal of alternating current frequencies. Where the exported sampling frequency differed from 1000 Hz, cubic spline re-sampling to this frequency was used. Although low-pass filtering with 100 Hz cut-off was applied (see subsequent Supplementary Figure 1), the 1000 Hz frequency was used for the purposes of obtaining interval measurements (in representative beats) with 1 millisecond precision. LSB – least significant bit, s -seconds.

Supplementary Figure 1

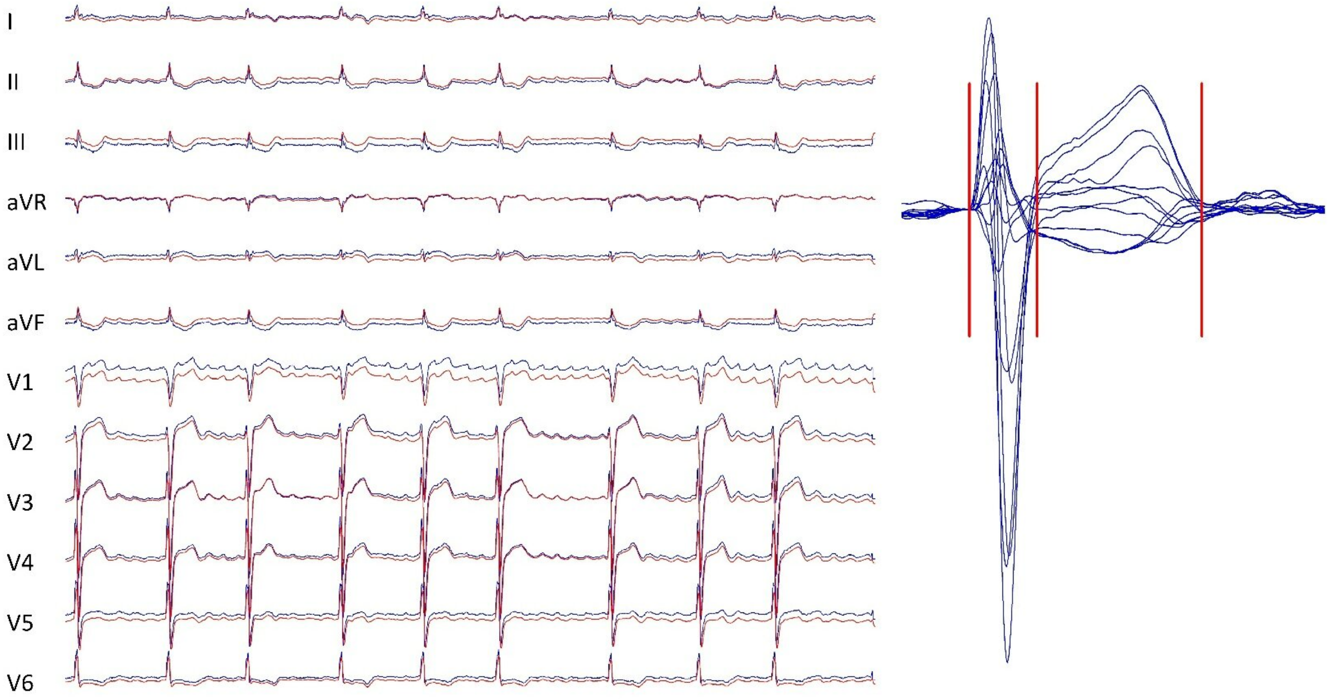

Example of ECG pre-processing shown in a case of an atrial fibrillation patient. The left panel shows the original ECG signal in blue superimposed by filtered signals in red. The filtering was performed in two steps: (a) A low pass infinite-impulse-response Butterworth filter with 100 Hz cut-off frequency was used to eliminate high-frequency noise (it also harmonised the frequency contents of all the study ECGs). (b) Subsequently, for each detected QRS complex (combination of maximum absolute amplitudes in the native signal and its derivative) a window of preceding 100 ms was used to identify the point with minimum standard deviation across all leads. These points identified baseline wander nodes and a cubic spline interpolation across these nodes was subtracted from the filtered signal to remove baseline wander.

The right panel shows representative beatforms derived, for each ECG lead, by obtaining sample by sample medians across all superimposed QRS complexes. These representative beatforms of all 12 leads were superimposed on the same isoelectric axis and the resulting image was used to detect the global QRS onset and offset as well as the T wave offset (red vertical lines).

Supplementary Table 2

Kendall's τ coefficients
between QRS micro-fragmentation and other risk factors

	EU-CERT-ICD		VA Washington		Whitehall	
	τ	p-value	τ	p-value	τ	p-value
Age [years]	0.098	<0.001	0.049	0.046	0.042	<0.001
Heart rate [bpm]	-0.024	0.107	-0.011	0.658	-0.058	<0.001
LVEF [%]	-0.104	<0.001				
QRS duration [ms]	0.358	<0.001	0.338	<0.001	0.304	<0.001
QTc [ms]	0.240	<0.001	0.156	<0.001	0.071	<0.001
TCRT [deg]	0.087	<0.001	0.141	<0.001	0.060	<0.001

Bpm – beats per minute, deg – degrees, LVEF – left ventricular ejection fraction, ms – milliseconds, TCRT – total cosine R to T

Supplementary Figure 2

EU-CERT-ICD

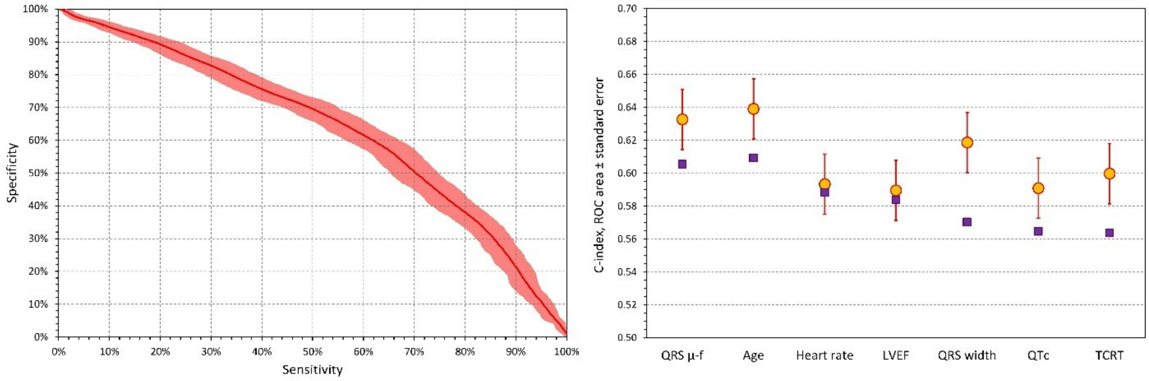

VA WASHINGTON

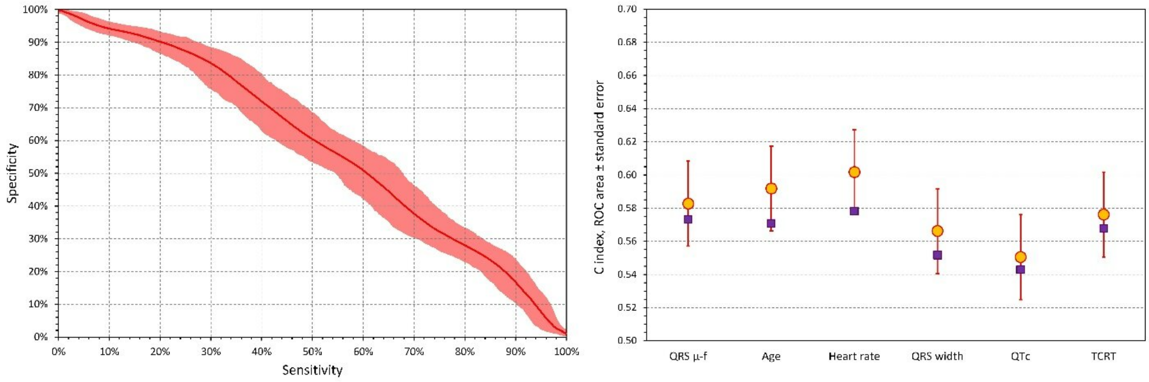

WHITEHALL II

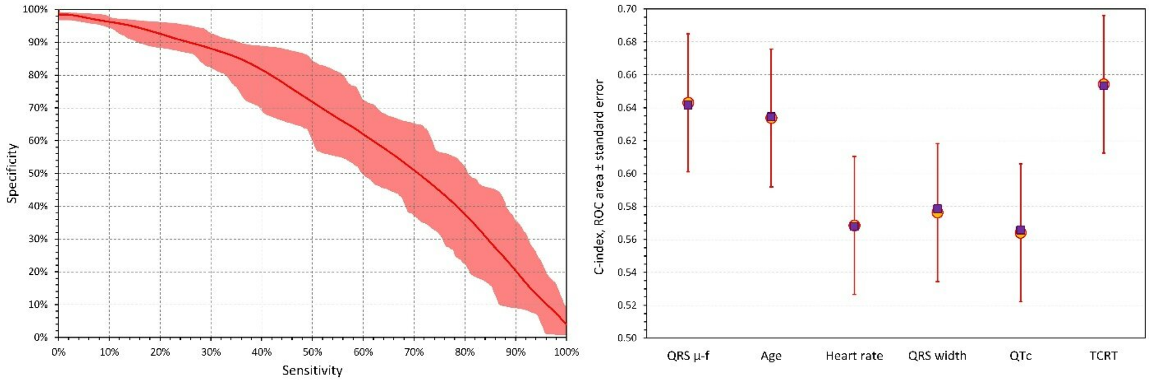

For each of the investigated populations, the left panels show QRS micro-fragmentation receiver operator characteristic (ROC) for events during the complete follow-up, together with its 90% confidence band obtained by bootstrap with 1000 repetitions. The right panels show the areas under the ROC curve for different continuous risk predictor together with their standard errors (red marks) and the Harrell's C-index values (dark violet marks). Note the differences between the ROC areas and the C-index values due to follow-up influence.

LVEF – left ventricular ejection fraction, TCRT – total cosine R to T, µ-f – QRS micro-fragmentation.

Supplementary Table 3

Multivariable Harrell's C-index statistics

	Model of Score 1	Model of Score 2
**EU-CERT-ICD**		
Age [years]	0.036618	0.033671
Heart rate [bpm]	0.016280	0.017610
LVEF [%]	-0.028609	-0.025182
QTc [ms]	0.003667	0.001400
TCRT [deg]	0.005332	0.005427
$\log_2$(QRS μ-fragmentation)		0.407542
Cox regression χ^2	104.2	138.7
Area under the ROC curve	0.698	0.715
Harrell's C-index	0.652	0.667
**VA Washington**		
Age [years]	0.031944	0.032516
Heart rate [bpm]	0.010328	0.011130
QTc [ms]	0.005928	0.004068
TCRT [deg]	0.006194	0.005320
$\log_2$(QRS μ-fragmentation)		0.260371
Cox regression χ^2	35.0	41.2
Area under the ROC curve	0.648	0.657
Harrell's C-index	0.615	0.627
**Whitehall II**		
Age [years]	0.072306	0.067307
TCRT [deg]	0.013992	0.012176
$\log_2$(QRS μ-fragmentation)		0.448389
Cox regression χ^2	40.4	47.5
Area under the ROC curve	0.714	0.739
Harrell's C-index	0.702	0.728

For each of the investigated population, multivariable Cox regression model involving the continuous variables as shown in Table 2 of the article was computed without (Model of Score 1) and with (Model of Score 2) QRS micro-fragmentation. The table shows the resulting beta coefficients (log hazard ratios) assigned to each of the variables that were retained during the backwards stepwise elimination for Model Score 1. The Model score 2 shows the Cox regression beta coefficients after QRS micro-fragmentation was added to the variables of Model Score 1. The beta coefficients were used as weights of the variables to obtain weighted average risk scores. The blue lines of the table show the overall χ^2 statistics of the Cox regression models that provided the beta coefficients, areas under the receiver operator characteristic of the derived risk scores (for events across the complete follow-up), and the Harrell's C-index values of the derived risk scores. Note that in all three populations, the inclusion of QRS micro-fragmentation increased the χ^2 statistics, the area under the receiver operator characteristic, and the C-index statistics.

bpm – beats per minute, deg – degrees, ms – milliseconds, ROC – receiver operator characteristic, TCRT – total cosine R to T, μ-fragmentation – micro-fragmentation.

Supplementary Figure 3

EU-CERT-ICD

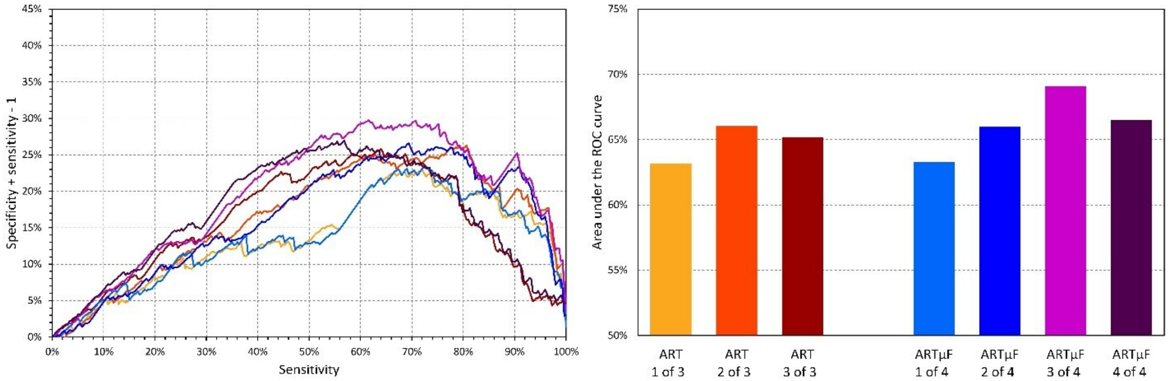

VA WASHINGTON

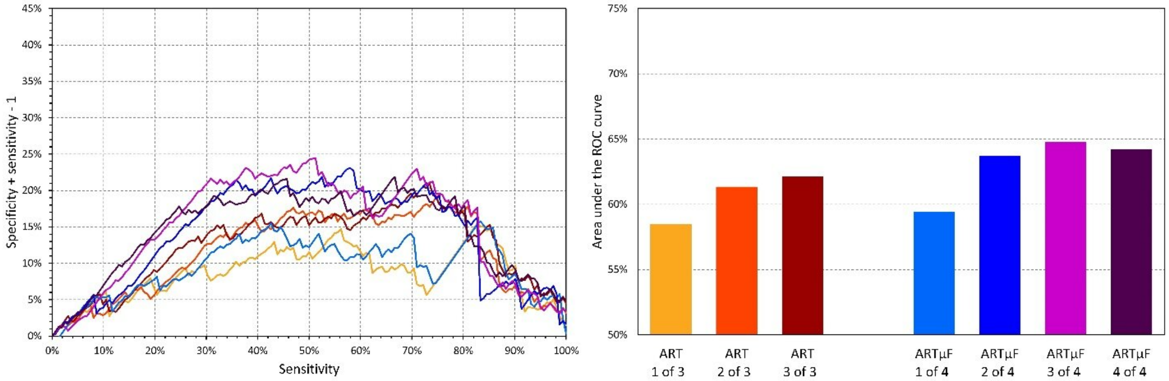

WHITEHALL II

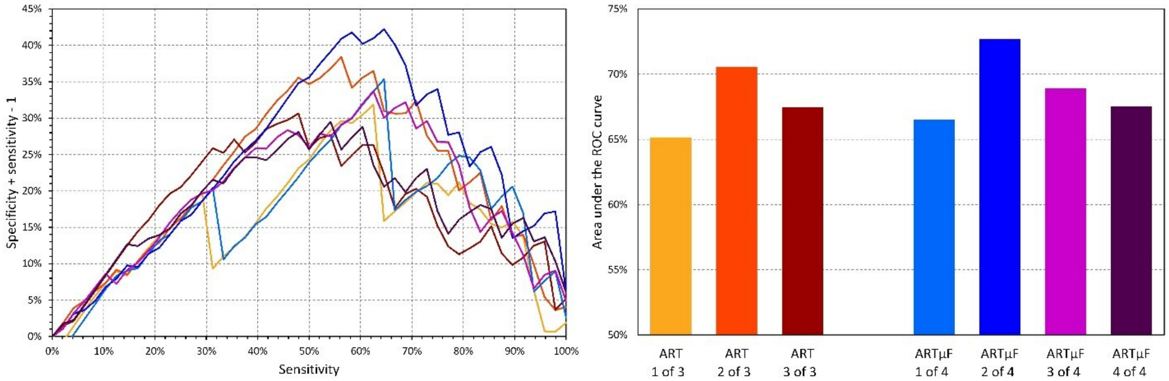

For each of the investigated populations, the left panels show multifactorial receiver operator characteristic (ROC) for events during the complete follow-up. The right panels show the areas under these ROC curves. Two groups of ROC curves are shown: Those labelled ART combined age, heart rate, and total cosine R to T; those labelled ARTμF included also QRS micro-fragmentation. Within each group, the ROC curves differed by the definition of true/false positive/negative: as the dichotomies of the risk factors involved were varied, for each of their combinations, positive cases were defined as those subjects for whom the values of 1, 2, 3, or 4 risk factors were above the given dichotomy. To ease the comparison of the ROC curves, their values are shown above the 50% identity line, i.e., the panels on the left show the dependency of (specificity+sensitivity-1) on sensitivity. The colours of these curves correspond to the bar graphs.

Supplementary Table 4

Association between mortality and continuous values of risk factors
in aetiology sub-groups of EU-CERT-ICD

	Univariable analysis			Multivariable analysis		
	Wald	p-value	HR (95% CI)	Wald	p-value	HR (95% CI)
**Ischaemic heart disease**						
Age	42.3	<0.001	1.056 (1.038 - 1.073)	30.1	<0.001	1.048 (1.031 - 1.066)
Heart rate [bpm]	16.1	<0.001	1.017 (1.009 - 1.026)	11.0	0.001	1.015 (1.006 - 1.023)
LVEF [%]	18.0	<0.001	0.958 (0.940 - 0.977)	6.83	0.009	0.972 (0.952 - 0.993)
QRS duration [ms]	18.6	<0.001	1.010 (1.005 - 1.014)	5.55	0.018	0.992 (0.985 - 0.999)
QTc [ms]	16.9	<0.001	1.007 (1.004 - 1.011)	4.11	0.043	1.005 (1.000 - 1.009)
TCRT [deg]	25.2	<0.001	1.012 (1.007 - 1.017)	12.8	<0.001	1.010 (1.004 - 1.015)
$\log_2$(QRS µ-fragmentation)	21.5	<0.001	1.568 (1.297 - 1.897)	13.5	<0.001	1.533 (1.220 - 1.926)
**Non-ischaemic heart disease**						
Age	6.58	0.01	1.023 (1.005 - 1.042)	13.4	<0.001	1.025 (1.012 - 1.039)
Heart rate [bpm]	18.0	<0.001	1.027 (1.014 - 1.039)			
LVEF [%]	13.8	<0.001	0.951 (0.926 - 0.976)	6.27	0.012	0.965 (0.938 - 0.992)
QRS duration [ms]	13.2	<0.001	1.012 (1.006 - 1.019)			
QTc [ms]	8.23	0.004	1.007 (1.002 - 1.013)			
TCRT [deg]	5.90	0.015	1.010 (1.002 - 1.018)			
$\log_2$(QRS µ-fragmentation)	24.6	<0.001	2.077 (1.556 - 2.772)	21.1	<0.001	1.986 (1.482 - 2.662)

Multivariable analysis used backwards stepwise elimination. In addition to hazard ratios, Wald statistics are shown. QRS micro-fragmentation was used after logarithmic transformation with base 2 – hazard ratios correspond to value increases by a factor of 2.

CI – confidence interval, bpm- beats per minute, deg – degrees, HR – hazard ratio, LVEF – left ventricular ejection fraction, ms – milliseconds, TCRT – total cosine R to T, µ-fragmentation – micro-fragmentation.

Supplementary Table 5

Characteristics of EU-CERT-ICD population per contributing centre

	Survivors	Non-survivors	p-value
**BASEL**			
N	423	65	
Age [years]	64 (54 - 70)	69 (62 - 74)	0.0001
Heart rate [bpm]	66 (58.9 - 77.4)	75 (65.1 - 83.2)	0.0050
LVEF [%]	27 (24 - 33)	25 (22 - 30)	0.0012
QRS duration [ms]	124 (109 - 156)	152 (120 - 174)	<0.0001
QTc [ms]	447 (424 - 472)	458 (432 - 494)	0.0140
TCRT [deg]	149 (102 - 165)	160 (136 - 165)	0.0278
QRS µ-fragmentation [%]	3.133 (2.289 - 4.37)	4.070 (2.748 - 6.747)	<0.0001
**GÖTTINGEN**			
N	334	107	
Age [years]	67 (57 - 74)	71 (67 - 77)	<0.0001
Heart rate [bpm]	70.7 (62.8 - 80.6)	73.5 (66.3 - 84.8)	0.0268
LVEF [%]	25 (20 - 30)	25 (20 - 30)	0.1850
QRS duration [ms]	134 (115 - 163)	147 (120 - 169)	0.0322
QTc [ms]	435 (413 - 459)	444 (421 - 471)	0.0390
TCRT [deg]	153 (118 - 166)	158 (145 - 168)	0.0029
QRS µ-fragmentation [%]	3.211 (2.451 - 4.543)	4.186 (3.186 - 5.203)	<0.0001
**LEUVEN**			
N	327	34	
Age [years]	61 (53 - 69)	65 (56 - 70)	0.1538
Heart rate [bpm]	65.6 (57.1 - 75.2)	64.3 (59.1 - 77.3)	0.5496
LVEF [%]	27 (20 - 33)	25 (20 - 30)	0.1687
QRS duration [ms]	137 (116 - 165)	146 (127 - 174)	0.1730
QTc [ms]	449 (425 - 478)	466 (425 - 495)	0.1764
TCRT [deg]	151 (120 - 165)	164 (145 - 169)	0.0050
QRS µ-fragmentation [%]	3.242 (2.467 - 4.812)	4.246 (3.015 - 5.235)	0.0717
**OULU**			
N	30	2	
Age [years]	59 (51 - 65)	69 , 71	
Heart rate [bpm]	71.7 (59.1 - 82.2)	70.6 , 74.4	
LVEF [%]	30 (26 - 35)	21 , 26	
QRS duration [ms]	142 (112 - 155)	128 , 168	
QTc [ms]	453 (428 - 471)	414 , 469	
TCRT [deg]	165 (152 - 170)	169 , 171	
QRS µ-fragmentation [%]	3.375 (2.55 - 4.932)	3.460 , 6.036	

	Survivors	Non-survivors	p-value
**UTRECHT**			
N	540	86	
Age [years]	64 (57 - 72)	68 (60 - 74)	0.0313
Heart rate [bpm]	70.5 (61.4 - 81.6)	73.5 (66.4 – 87.0)	0.0204
LVEF [%]	25 (20 - 29)	20 (18 - 26)	0.0001
QRS duration [ms]	125 (112 - 154)	144 (123 - 170)	<0.0001
QTc [ms]	437 (416 - 462)	456 (437 - 489)	<0.0001
TCRT [deg]	150 (119 - 164)	159 (139 - 167)	0.0056
QRS µ-fragmentation [%]	3.224 (2.367 - 4.49)	4.271 (2.845 - 5.708)	0.0001

For individual centres of EU-CERT-ICD population, the table shows medians and inter-quartile ranges and their comparison between 5-year survivors and non-survivors. Non-parametric Mann-Whitney p-values are shown. The comparisons were omitted for the Oulu centre since only 2 non-survivors were contributed by the centre (instead of median and inter-quartile ranges, both values are shown for Oulu centre non-survivors).

Interestingly, when non-parametric Kruskal-Wallis one-way analysis of variance was used to test that the distribution of the risk factors shown is the same across centres, the distributions of all variables with the exception of QRS micro-fragmentation were found highly significantly different between centres ($p < 0.0001$ for age, heart rate, LVEF, QRS duration, and QTc interval; $p = 0.0007$ for TCRT). However, no differences were found between the distributions of QRS micro-fragmentation ($p = 0.4173$).

bpm – beats per minute, deg – degrees, ms – milliseconds, TCRT – total cosine R to T, µ-fragmentation – micro-fragmentation.

Supplementary Table 6

Association between mortality and continuous values of risk factors in contributing centres of EU-CERT-ICD

	Univariable analysis			Multivariable analysis		
	Wald	p-value	HR (95% CI)	Wald	p-value	HR (95% CI)
**BASEL**						
Age [years]	17.0	<0.001	1.057 (1.030 - 1.086)	12.9	<0.001	1.052 (1.023 - 1.082)
Heart rate [bpm]	10.2	0.001	1.023 (1.009 - 1.038)	11.0	0.001	1.026 (1.011 - 1.042)
LVEF [%]	10.5	0.001	0.948 (0.918 - 0.979)	5.66	0.017	0.953 (0.916 - 0.992)
QRS duration [ms]	18.7	<0.001	1.016 (1.009 - 1.024)			
$\log_2$(QRS µ-fragmentation)	22.6	<0.001	2.113 (1.552 - 2.877)	14.2	<0.001	1.827 (1.336 - 2.499)
**GÖTTINGEN**						
Age [years]	23.5	<0.001	1.055 (1.033 - 1.079)	17.8	<0.001	1.05 (1.026 - 1.074)
Heart rate [bpm]	5.68	0.017	1.015 (1.003 - 1.027)	7.61	0.006	1.017 (1.005 - 1.030)
LVEF [%]	2.74	0.098	0.978 (0.954 - 1.004)			
QRS duration [ms]	3.02	0.082	1.005 (0.999 - 1.011)			
$\log_2$(QRS µ-fragmentation)	12.0	0.001	1.660 (1.247 - 2.211)	7.84	0.005	1.552 (1.141 - 2.111)
**LEUVEN**						
Age [years]	1.45	0.229	1.020 (0.988 - 1.054)			
Heart rate [bpm]	2.84	0.092	1.022 (0.996 - 1.048)			
LVEF [%]	2.78	0.095	0.968 (0.932 - 1.006)	2.78	0.095	0.968 (0.932 - 1.006)
QRS duration [ms]	0.94	0.332	1.005 (0.995 - 1.016)			
$\log_2$(QRS µ-fragmentation)	1.40	0.237	1.324 (0.831 - 2.109)			
**UTRECHT**						
Age [years]	2.00	0.158	1.015 (0.994 - 1.036)			
Heart rate [bpm]	4.72	0.030	1.013 (1.001 - 1.025)			
LVEF [%]	5.90	0.015	0.959 (0.927 - 0.992)	5.19	0.023	0.961 (0.929 - 0.994)
QRS duration [ms]	7.32	0.007	1.010 (1.003 - 1.017)			
$\log_2$(QRS µ-fragmentation)	7.15	0.007	1.486 (1.112 - 1.986)	6.44	0.011	1.465 (1.091 - 1.968)

Multivariable analysis used backwards stepwise elimination. In addition to hazard ratios, Wald statistics are shown. QRS micro-fragmentation was used after logarithmic transformation with base 2 – hazard ratios correspond to value increases by a factor of 2.

CI – confidence interval, bpm- beats per minute, deg – degrees, HR – hazard ratio, LVEF – left ventricular ejection fraction, ms – milliseconds, µ-fragmentation – micro-fragmentation.

Supplementary Figure 4

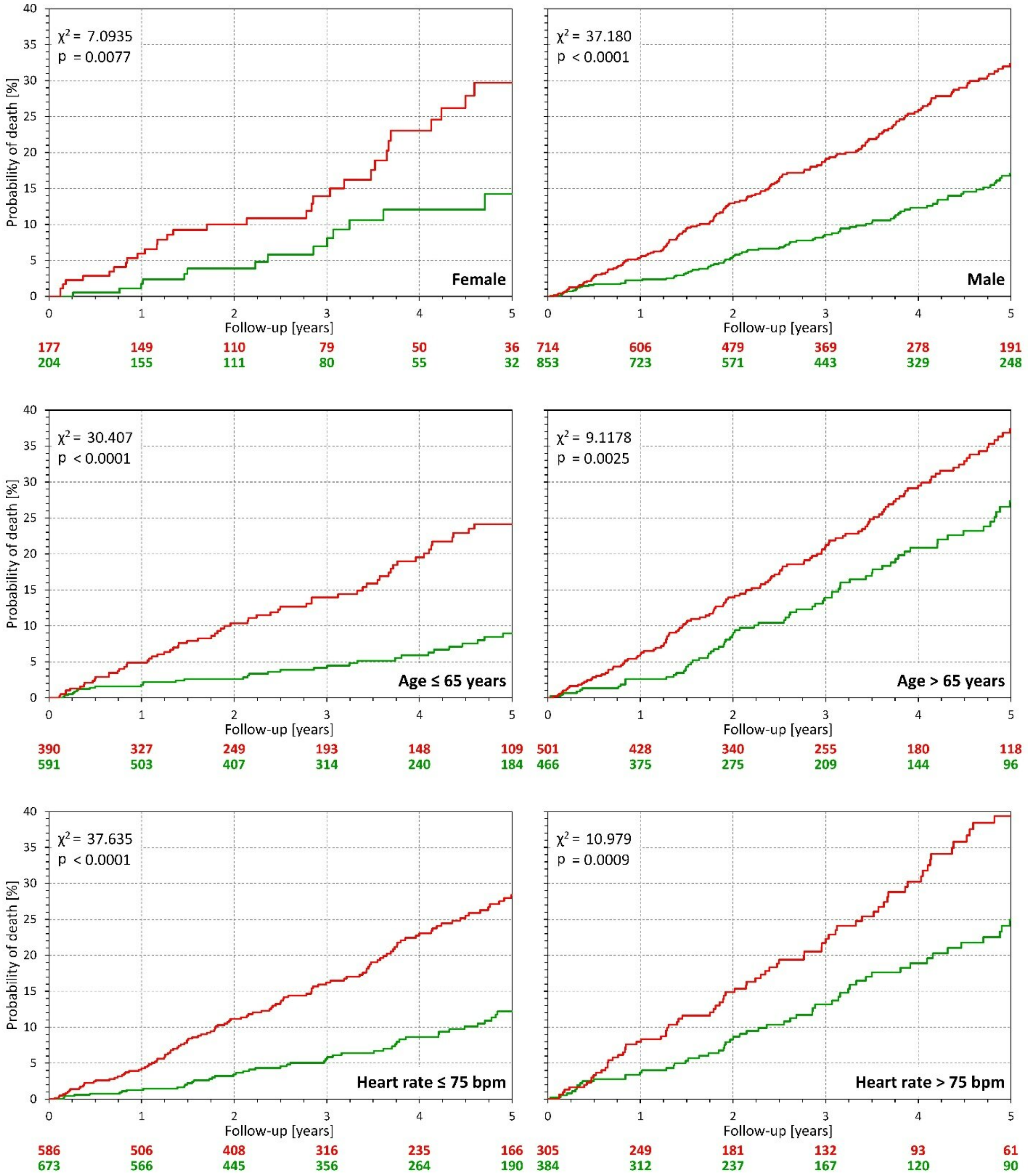

Individual panels of the figure show survival differences stratified by QRS micro-fragmentation ≤ 3.5% (green lines) and > 3.5% (red lines) in sub-populations of the EU-CERT-ICD data defined by sex (top row); by age dichotomised at 65 years (middle row), and by heart rate dichotomised at 75 beats per minute (bottom row). In each panel, the χ^2 statistics is shown together with the corresponding p-value (log-rank test). Numbers of patients at risk in the different strata are shown below the panels in colours corresponding to the Kaplan-Meier survival curves.

Supplementary Figure 5

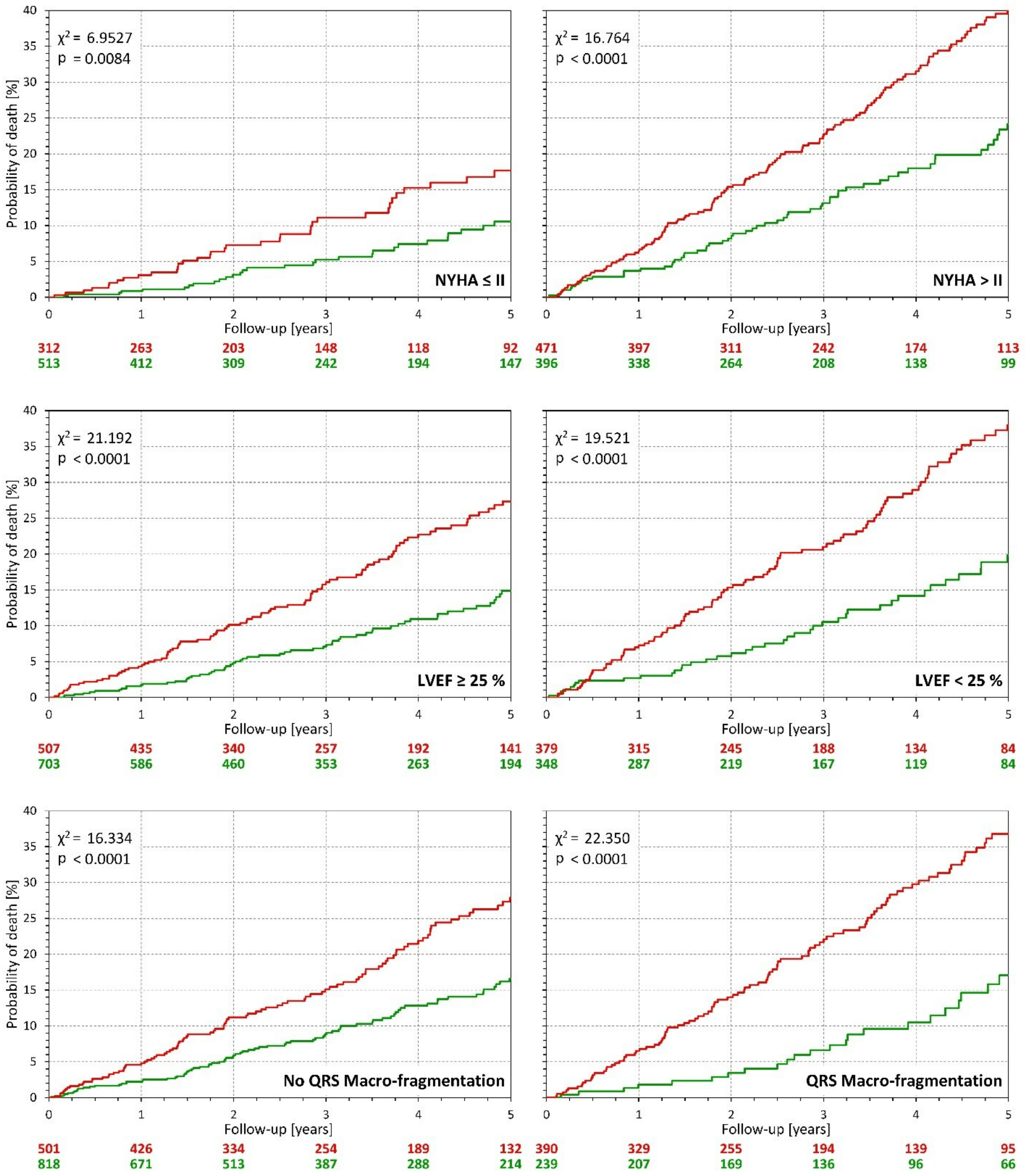

Individual panels of the figure show survival differences stratified by QRS micro-fragmentation ≤ 3.5% (green lines) and > 3.5% (red lines) in sub-populations of the EU-CERT-ICD data defined by New York Heart Association class (NYHA) assessed at ICD implantation and divided into classes I+II and III+IV (top row); by left ventricular ejection fraction (LVEF) dichotomised at 25% (middle row); and by the presence or absence of visible QRS complex macro-fragmentation (bottom row). In each panel, the χ^2 statistics is shown together with the corresponding p-value (log-rank test). Numbers of patients at risk in the different strata are shown below the panels in colours corresponding to the Kaplan-Meier survival curves (small number of cases with missing data excluded).

Supplementary Figure 6

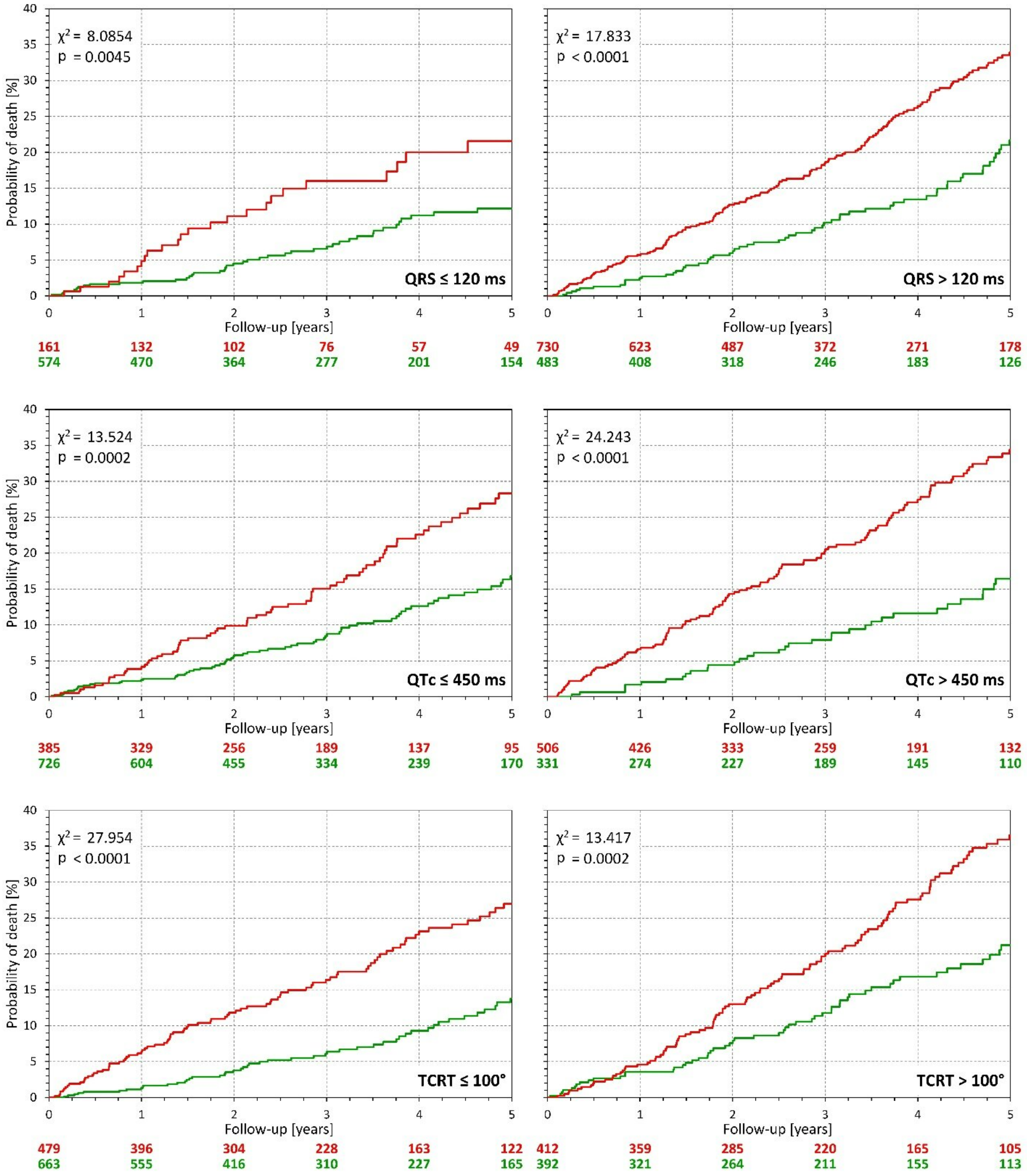

Individual panels of the figure show survival differences stratified by QRS micro-fragmentation ≤ 3.5% (green lines) and > 3.5% (red lines) in sub-populations of the EU-CERT-ICD data defined by QRS duration dichotomised at 120 ms (top row); by QTc interval dichotomised at 450 ms (middle row), and by the total cosine R to T (TCRT) dichotomised at 100° (bottom row). In each panel, the χ^2 statistics is shown together with the corresponding p-value (log-rank test). Numbers of patients at risk in the different strata are shown below the panels in colours corresponding to the Kaplan-Meier survival curves (small number of cases with missing data excluded).

Supplementary Figure 7

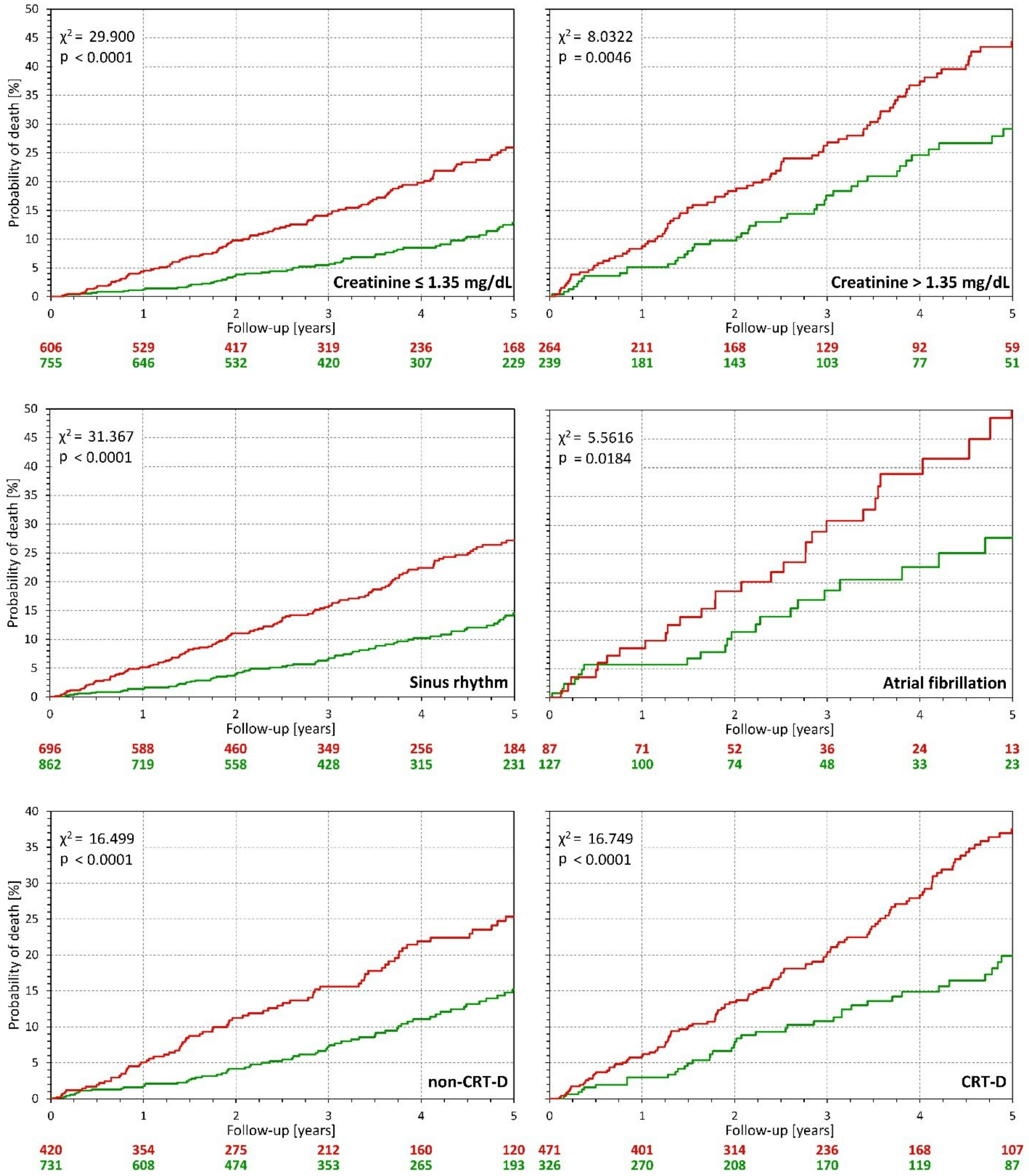

Individual panels of the figure show survival differences stratified by QRS micro-fragmentation ≤ 3.5% (green lines) and > 3.5% (red lines) in sub-populations of the EU-CERT-ICD data defined by creatinine plasma levels dichotomised at 1.35 mg/dL (top row); by the rhythm of the analysed electrocardiogram (middle row – see Supplementary Table 6 for further details); and by the distinction on whether the patients were, for clinical reasons, implanted with a cardiac resynchronisation defibrillator or with a device without the resynchronisation function (bottom row). In each panel, the χ^2 statistics is shown together with the corresponding p-value (log-rank test). Numbers of patients at risk in the different strata are shown below the panels in colours corresponding to the Kaplan-Meier survival curves (small number of cases with missing data excluded).

Supplementary Figure 8

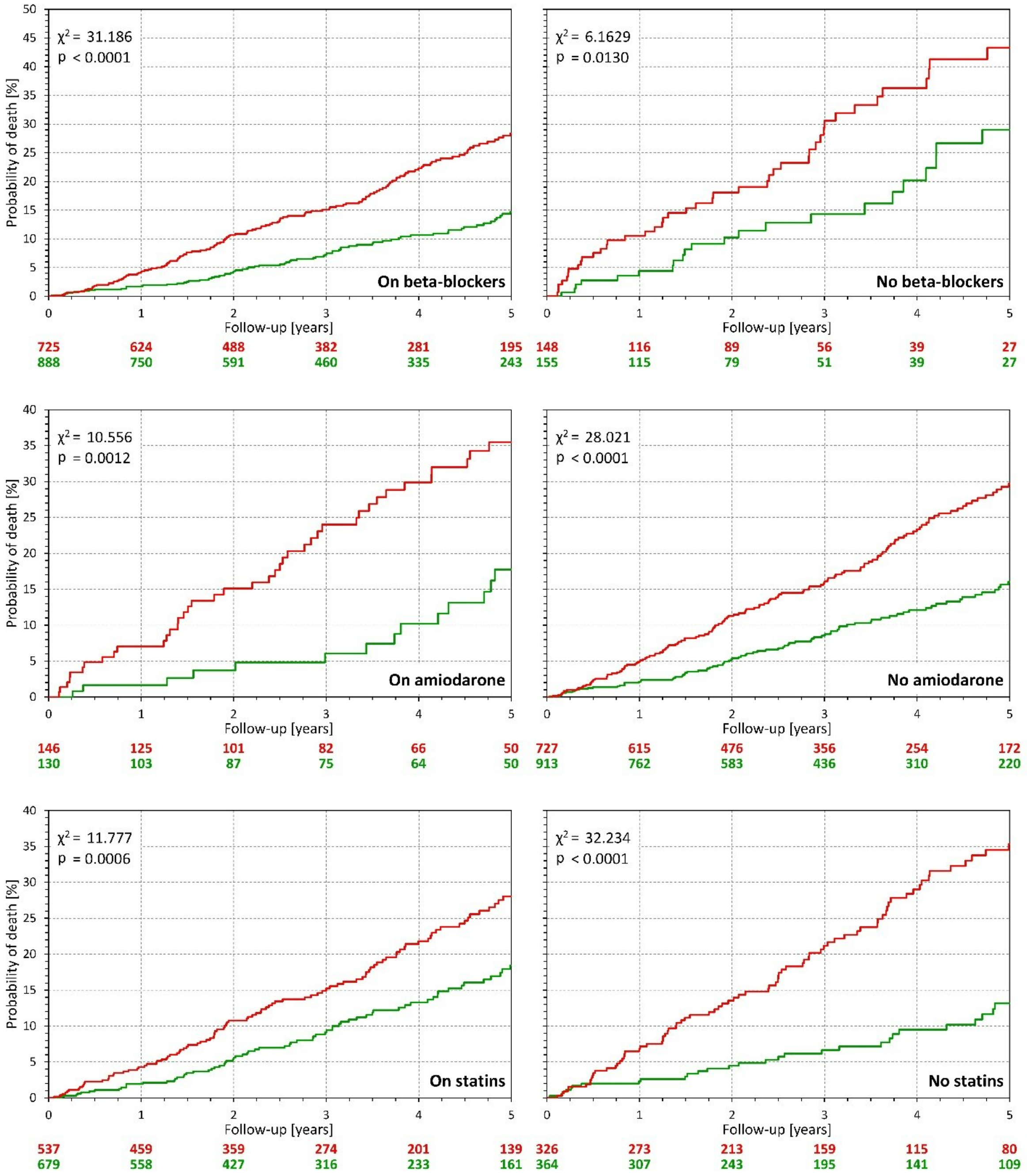

Individual panels of the figure show survival differences stratified by QRS micro-fragmentation ≤ 3.5% (green lines) and > 3.5% (red lines) in sub-populations of the EU-CERT-ICD data defined by intention to treat by beta-blockers (top row); amiodarone (middle row), and statins (bottom row). In each panel, the χ^2 statistics is shown together with the corresponding p-value (log-rank test). Numbers of patients at risk in the different strata are shown below the panels in colours corresponding to the Kaplan-Meier survival curves (small number of cases with missing data excluded).

Supplementary Table 7

Association between mortality and continuous values of risk factors in EU-CERT-ICD in sinus rhythm and in atrial fibrillation

	Univariable analysis			Multivariable analysis†		
	Wald	p-value	HR (95% CI)	Wald	p-value	HR (95% CI)
**Patients in sinus rhythm** (n=1558)						
Age [years]	28.3	<0.001	1.039 (1.024 - 1.054)	20.0	<0.001	1.033 (1.019 - 1.048)
Heart rate [bpm]	26.9	<0.001	1.022 (1.014 - 1.031)	20.7	<0.001	1.020 (1.011 - 1.029)
LVEF [%]	24.5	<0.001	0.955 (0.937 - 0.972)	8.86	0.003	0.969 (0.950 - 0.989)
QRS duration [ms]	20.7	<0.001	1.011 (1.006 - 1.015)			
QTc [ms]	11.7	0.001	1.006 (1.003 - 1.010)			
TCRT [deg]	20.2	<0.001	1.011 (1.006 - 1.015)	5.81	0.016	1.006 (1.001 - 1.011)
$\log_2$(QRS µ-fragmentation)	27.9	<0.001	1.691 (1.391 - 2.055)	15.9	<0.001	1.521 (1.237 - 1.869)
**Patients in atrial fibrillation** (n=214)						
Age [years]	1.58	0.209	1.022 (0.988 - 1.058)			
Heart rate [bpm]	0.77	0.379	1.008 (0.990 - 1.026)			
LVEF [%]	3.35	0.067	0.964 (0.928 - 1.003)			
QRS duration [ms]	2.98	0.085	1.007 (0.999 - 1.016)			
QTc [ms]	5.08	0.024	1.006 (1.001 - 1.012)			
TCRT [deg]	3.60	0.058	1.011 (1.000 - 1.022)	3.97	0.046	1.011 (1.000 - 1.022)
$\log_2$(QRS µ-fragmentation)	10.7	0.001	1.692 (1.235 - 2.318)	14.0	<0.001	1.898 (1.358 - 2.654)

Of the 1948 patients of the EU-CERT-ICD data collection, 1558 had the ECG classified as sinus rhythm, 214 were in atrial fibrillation, 123 had the rhythm classified as “other” (trigeminy, frequent ectopic beats, atrial flutter, paced rhythm, etc.) and 53 patients had the rhythm unclassified. The analyses shown in this table show only patients with confirmed sinus rhythm and confirmed atrial fibrillation.

Multivariable analysis used backwards stepwise elimination. In addition to hazard ratios, Wald statistics are shown. QRS micro-fragmentation was used after logarithmic transformation with base 2 – hazard ratios correspond to value increases by a factor of 2.

CI – confidence interval, bpm- beats per minute, deg – degrees, HR – hazard ratio, LVEF – left ventricular ejection fraction, ms – milliseconds, µ-fragmentation – micro-fragmentation.

Supplementary Figure 9

EU-CERT-ICD

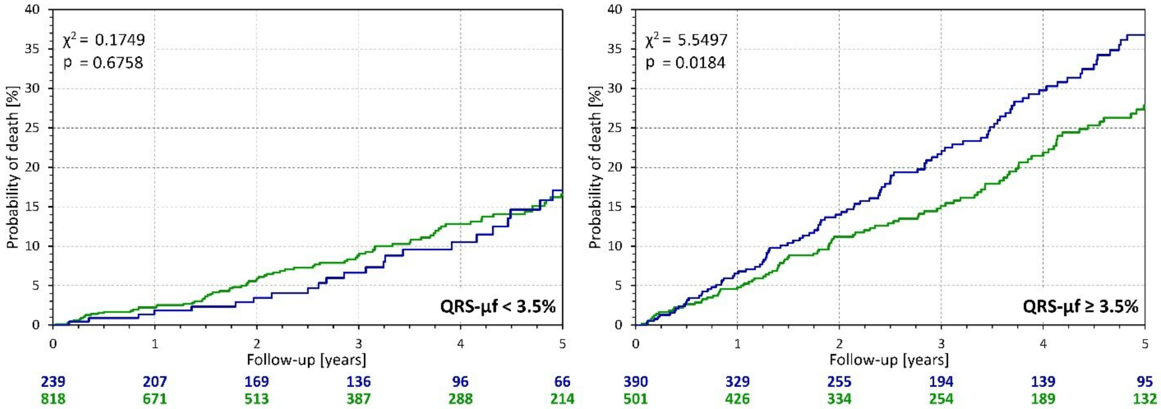

VA WASHINGTON

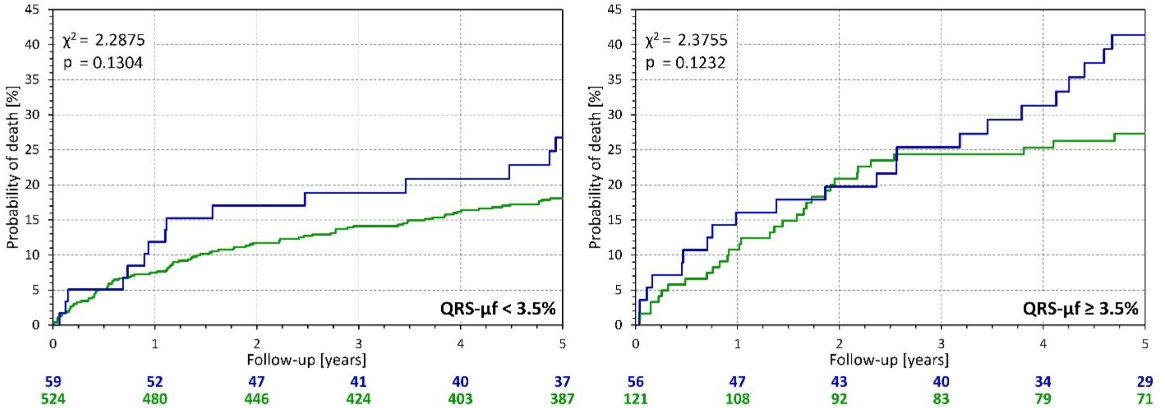

WHITEHALL II

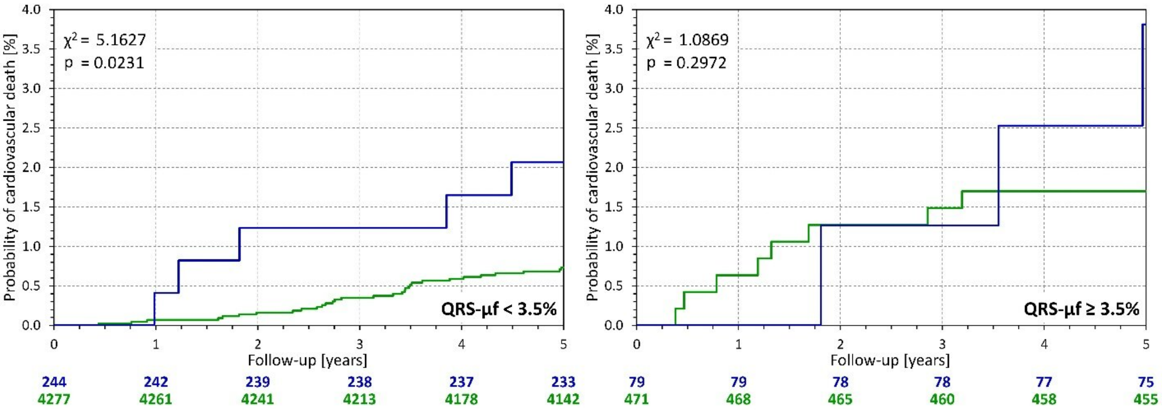

For each of the investigated populations, the panels show survival differences stratified by the presence (blue lines) and absence (green lines) of visible QRS macro-fragmentations in subpopulations with QRS micro-fragmentation < 3.5% (left panels) and ≥ 3.5% (right panels). In each panel, the χ^2 statistics is shown together with the corresponding p-value (log-rank test). Numbers of patients at risk in the different strata are shown below the panels in colours corresponding to the Kaplan-Meier survival curves.

Supplementary Figure 10

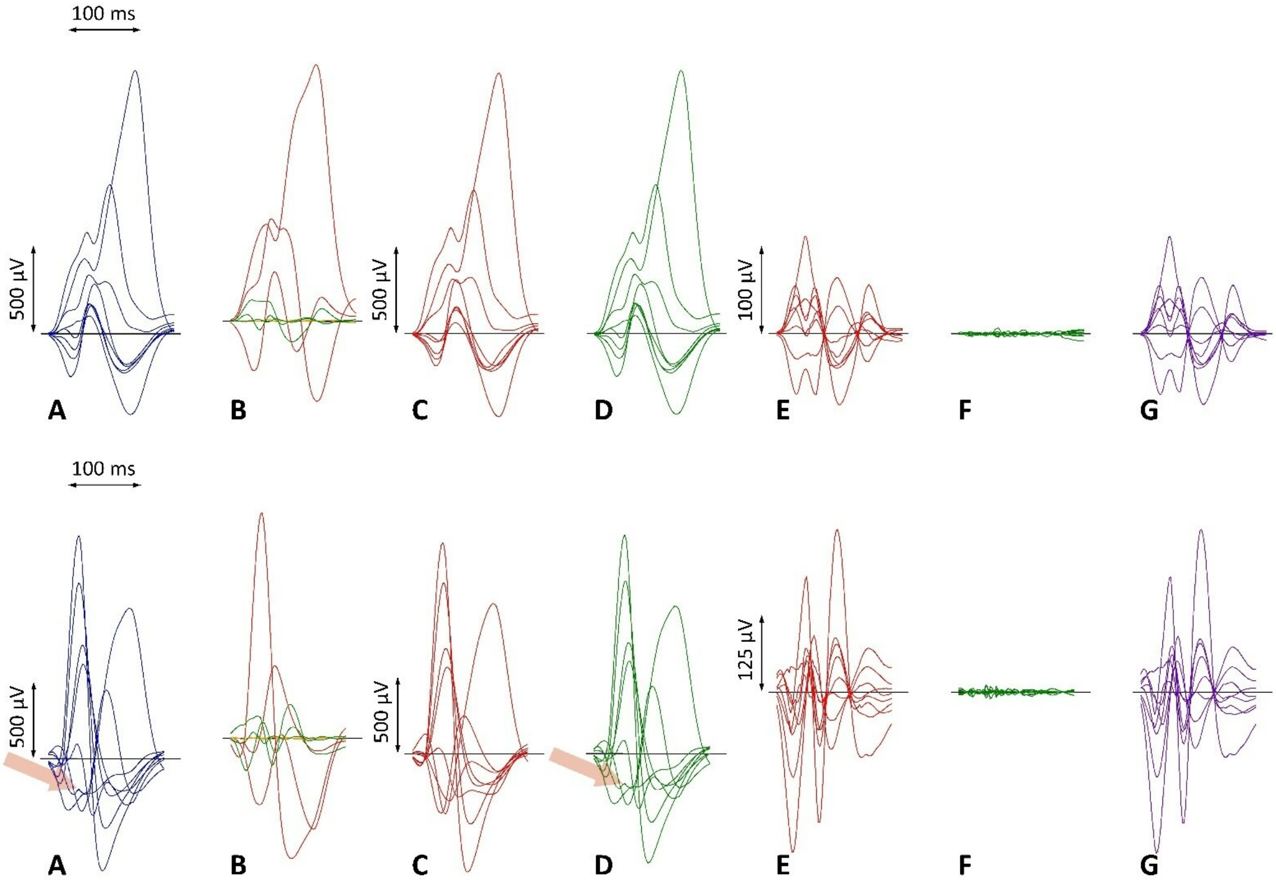

The panels of the Figure have the same meaning as the panels of Figure 1 of the main manuscript. The top and bottom rows correspond to 52-year and 67-year old male patients who died 4 years and 2 months later, respectively. Note that in the top row (QRS width of 172 ms), the clear macro-fragmentation of the QRS complex is reproduced in the reconstruction by the first 3 components (i.e., visible on panel **C**) and thus is present in the convolution of the 3-dimensional depolarisation vector. Other abnormalities of the ECG correspond to QRS micro-fragmentation of 9.429%. On the contrary, in the bottom row (QRS width of 158 ms) the macro-fractionation seen in lead V2 (arrow in panel **A**) is not reproduced in the 3-dimensional reconstruction but is present in the 6-dimensional reconstruction (arrow in panel **D**). This means that the abnormality of this macro-fragmentation is also present (but not clearly visible) in other leads and contributes to the QRS micro-fragmentation of 16.942%.

Supplementary Figure 11

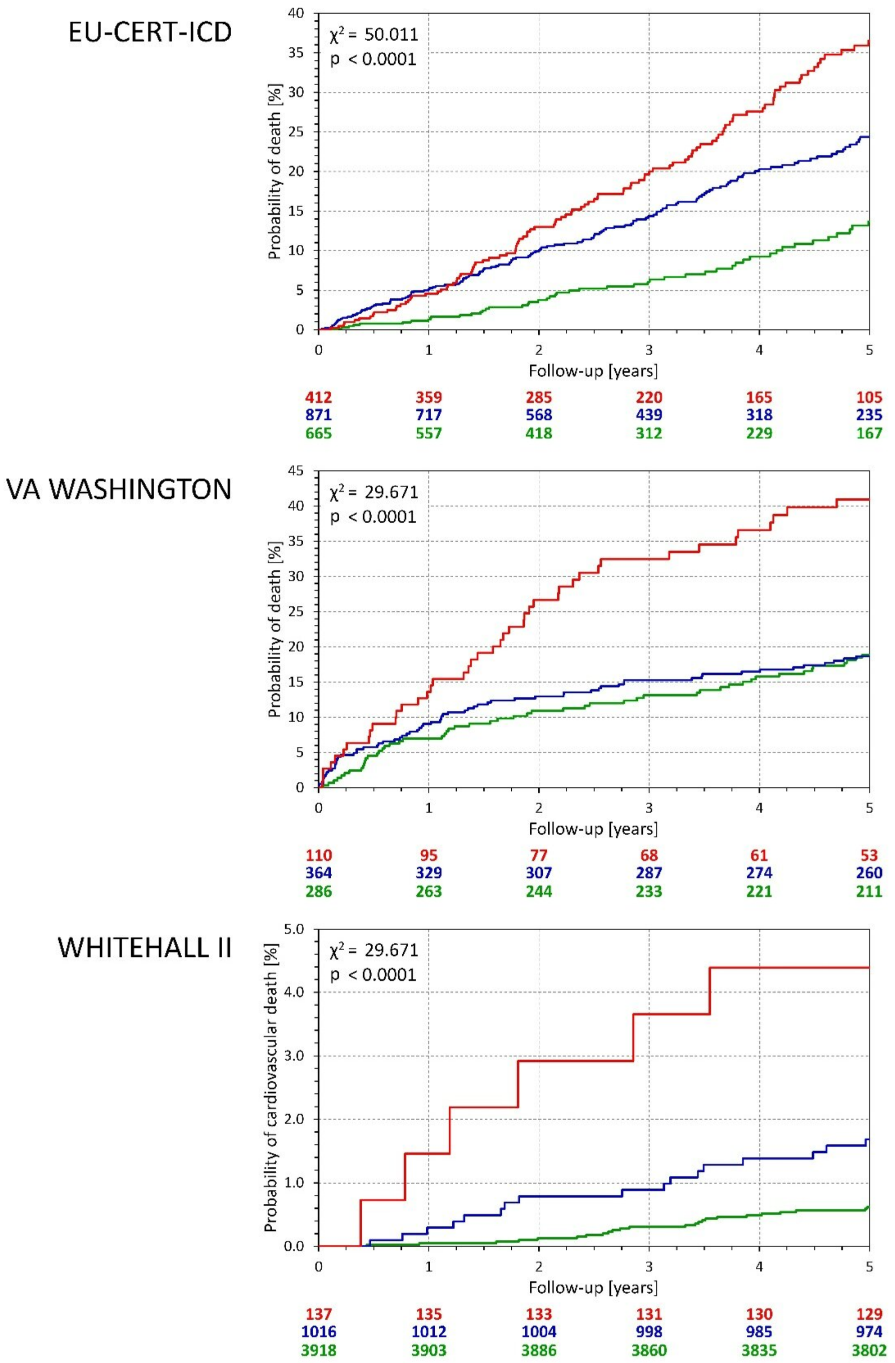

For each of the populations, the graphs show outcome probabilities in sub-groups stratified by a combination of QRS micro-fragmentation dichotomised at 3.5% and of total cosine R to T (TCRT) dichotomised at 100°. The green, blue, and red lines correspond to both factors normal, only one factor normal, and both factor abnormal, respectively. In each panel, the χ^2 statistics is shown together with the corresponding p-value (log-rank test). Numbers of patients at risk in the different strata are shown below the panels in colours corresponding to the Kaplan-Meier survival curves.
